# *Vibrio parahaemolyticus* Effector Proteins Suppress Inflammasome Activation by Interfering with Host Autophagy Signaling

**DOI:** 10.1371/journal.ppat.1003142

**Published:** 2013-01-24

**Authors:** Naomi Higa, Claudia Toma, Yukiko Koizumi, Noboru Nakasone, Toshitsugu Nohara, Junya Masumoto, Toshio Kodama, Tetsuya Iida, Toshihiko Suzuki

**Affiliations:** 1 Department of Molecular Bacteriology and Immunology, Graduate School of Medicine, University of the Ryukyus, Nishihara, Okinawa, Japan; 2 Department of Pathogenomics, Ehime University Graduate School of Medicine, Toon, Ehime, Japan; 3 International Research Center for Infectious Diseases, Research Institute for Microbial Diseases, Osaka University, Suita, Osaka, Japan; Purdue University, United States of America

## Abstract

Bacterial pathogens utilize pore-forming toxins or sophisticated secretion systems to establish infection in hosts. Recognition of these toxins or secretion system by nucleotide-binding oligomerization domain leucine-rich repeat proteins (NLRs) triggers the assembly of inflammasomes, the multiprotein complexes necessary for caspase-1 activation and the maturation of inflammatory cytokines such as IL-1β or IL-18. Here we demonstrate that both the NLRP3 and NLRC4 inflammasomes are activated by thermostable direct hemolysins (TDHs) and type III secretion system 1 (T3SS1) in response to *V. parahaemolyticus* infection. Furthermore, we identify T3SS1 secreted effector proteins, VopQ and VopS, which induce autophagy and the inactivation of Cdc42, respectively, to prevent mainly NLRC4 inflammasome activation. VopQ and VopS interfere with the assembly of specks in infected macrophages. These data suggest that bacterial effectors interfere with inflammasome activation and contribute to bacterial evasion from the host inflammatory responses.

## Author Summary

*V. parahaemolyticus* is Gram-negative pathogen that causes a food poisoning in human. To date, a number of bacterial factors that play a role in *V. parahaemolyticus* virulence have been characterized, yet little is known about the host factors contributing to the disease process and susceptibility to these pathogens. IL-1β, in addition to TNF-α, is thought to be involved in inflammatory responses and disease development during infection with the pathogen, but the mechanisms of IL-1β production remain poorly defined. In this work we found that both the NLRP3 and NLRC4 inflammasomes are activated by thermostable direct hemolysins (TDHs) and type III secretion system 1 (T3SS1) in response to *V. parahaemolyticus* infection. The activated inflammasomes then triggers the activation of caspase-1, a cysteine protease that is essential for IL-1β processing and release. Furthermore, we identified T3SS1 secreted effector proteins, VopQ and VopS, which prevent mainly NLRC4 inflammasome activation. VopQ and VopS induce autophagy and the inactivation of Rho GTPases, including Cdc42, respectively, and these cellular events interfere with the assembly of specks, the platform of inflammasome activation. Collectively, T3SS1 effector-based suppression of inflammasome activation may provide important insights into bacterial strategies for evading inflammasome-mediated host immune responses.

## Introduction

The innate immune responses play important roles in host defense against the infection by microbial pathogens. Several nucleotide-binding, oligomerization domain (NOD) leucine-rich repeat proteins (NLRs) and PYHIN proteins, such as NLRP1, NLRP3, NLRC4, AIM2, and IFI16, form inflammasomes: the multiprotein complexes that induce caspase-1 activation by functioning as sensors of pathogen-associated molecular patterns (PAMPs) or danger-associated molecular patterns (DAMPs) [1], [2], [3]. Inflammasome assembly is necessary for caspase-1 activation, and active caspase-1 then induces the processing of pro-IL-1β and pro-IL-18 and the secretion of mature active proinflammatory cytokines. Caspase-1 activation also triggers a rapid proinflammatory cell death known as pyroptosis. The NLRC4 inflammasome is activated in response to bacterial flagellin or rod protein, an essential component of the type III secretion system (T3SS) of Gram-negative bacteria. Flagellin that has been delivered into the cytoplasm of infected cells by the T3SS or type IV secretion system (T4SS) can bind to NAIP5 and facilitate NAIP5-NLRC4 interaction following the triggering of NLRC4 inflammasome assembly. On the other hand, rod protein such as PrgJ of *Salmonella enterica* delivered by the T3SS can bind to NAIP2 and promote NAIP2-NLRC4 interaction [4], [5]. The NLRP3 inflammasome is activated in response to a wide variety of stimuli, such as bacterial pore-forming toxins, ionophores, and noninfectious crystals or materials [1]. The stimulants trigger the cellular signals responsible for NLRP3 inflammasome activation, including a change in the intracellular potassium concentration, the generation of reactive oxygen species, lysosomal disruption or mitochondrial dysfunction [1]. A recent report has shown that oxidized mitochondrial DNA released from damaged mitochondria can bind NLRP3 and trigger inflammasome activation [6]. However, whether the released mitochondrial DNA is definitively triggered by the cellular signals for NLRP3 activation listed above remains to be elucidated.

Upon bacterial infection, the inflammasome is triggered as a proinflammatory response in host cells by sensing the pore-forming toxins or virulence-associated secretion systems. On the other hand, bacterial virulence strategies can interfere with essential components of immune signaling pathways such as NF-κB activation by secreting effectors that dampen cellular signals [7]. Also, bacteria use strategies that manipulate inflammasome activation, in most cases interfering with the production or recognition of bacterial ligands that trigger inflammasomes [3]. A recent study has shown that YopK, one of the T3SS effectors of *Yersinia pseudotuberculosis*, interacts with the T3SS translocon, thereby blocking the inflammasome from sensing the pathogen [8]. However, how bacterial pathogens interfere with inflammasome-mediated recognition of their secretion systems is largely unknown.

*Vibrio parahaemolyticus* is a gram-negative halophilic bacterium that is a leading cause of seafood-borne gastroenteritis [9]. As virulence factors for infection, this bacterium produces pore-forming toxins known as thermostable direct hemolysins (TDHs) and has two sets of T3SS: namely T3SS1 and T3SS-2 [10]. Histopathological study of the acute stage of infection with *V. parahaemolyticus* in humans has shown inflammatory responses with PMN infiltration, edema of the lamina propria and hemorrhage. Also, the secretions of TNF-α and IL-1β, two pro-inflammatory cytokines were markedly induced [11]. These results suggested that infection with *V. parahaemolyticus* induces inflammatory responses in the intestinal mucosa. However, how host cells recognize infection with *V. parahaemolyticus* and regulate the inflammatory responses remain largely unknown.

Since pore-forming toxins or virulence-associated secretion systems in bacterial infection mediate caspase-1 activation [12], [13], [14], we considered the possibility that *V. parahaemolyticus* might induce caspase-1 activation. Here we demonstrate that TDHs trigger NLRP3 inflammasome activation and that both the NLRP3 and NLRC4 inflammasomes are triggered in response to the T3SS1 in infected macrophages. We further show that two T3SS1-secreted effector proteins, VopQ and VopS, induce autophagy and Cdc42 inactivation, respectively; these processes consequently interfere mainly with NLRC4 inflammasome activation. Our data demonstrate that recognition of the activities of pore-forming toxins and the T3SS1 contribute to the host proinflammatory responses, and define the effector proteins that dampen the host responses by interfering with inflammasome activation. Preventing inflammasome activation by the T3SS1 effectors appears to be one strategy for bacterial evasion of the host proinflammatory responses.

## Results

### Infection with *V. parahaemolyticus* induces caspase-1 activation, IL-1β maturation and pyroptosis in macrophages via NLRP3 and NLRC4 inflammasomes

**Figure 1:**
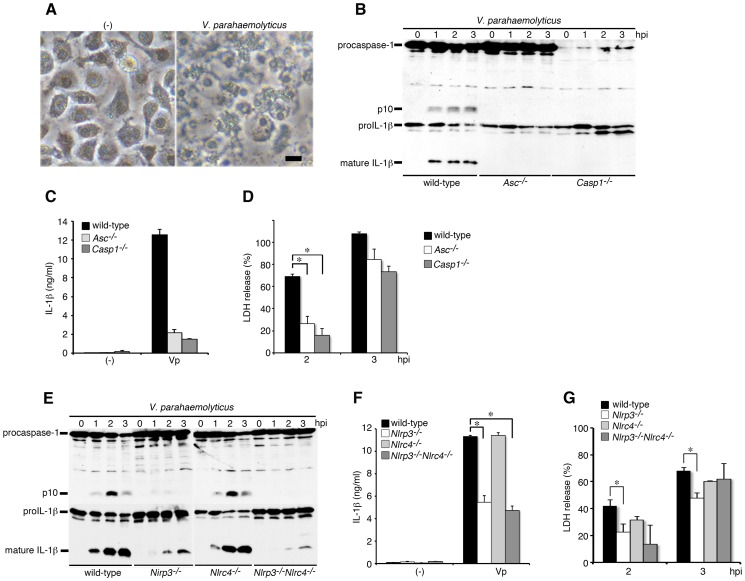
Infection with *V. parahaemolyticus* induces caspase-1 activation, IL-1β maturation and pyroptosis via NLRP3 and NLRC4 inflammasomes. BMMs from wild-type, ASC-deficient (*Asc* ^−/−^), caspase-1-deficient (*Casp1* ^−/−^), NLRP3-deficient (*Nlrp3* ^−/−^), NLRC4-deficient (*Nlrc4* ^−/−^), or NLRP3/NLRC4-double deficient (*Nlrp3* ^−/−^ *Nlrc4* ^−/−^) mice were primed with LPS (1 µg/ml; 3 hr) and infected with wild-type *V. parahaemolyticus* (Vp). **A.** Phase contrast images of wild-type BMMs either uninfected (-) or 3 hour post-infection (hpi). Representative damaged macrophages are shown. Bar, 20 µm. **B and E.** The activation of caspase-1 and IL-1β processing in infected BMMs were analyzed using immunoblotting with anti-caspase-1 or anti-IL-1β antibody. **C and F.** IL-1β secretion from the infected BMMs into the culture supernatants at 3 hpi. was analyzed using an ELISA. *p<0.05. **D and G.** Culture supernatants from infected BMMs were analyzed for LDH release. Data are presented as the means ± SD of triplicate samples. *p<0.05.

Mouse bone marrow-derived macrophages (BMMs) were primed with LPS to induce proIL-1β (35 kDa) expression and infected with a wild-type (WT) *V. parahaemolyticus* strain. We observed that infected BMMs underwent lytic cell death with membrane swelling by 3 hours post infection (hpi) (**Figure 1A**). Furthermore, infection with *V. parahaemolyticus* induced caspase-1 activation (production of p10 fragment by processing procaspase-1 (45 kDa)) and the processing (production of 17 kDa mature form)/release of proIL-1β (**Figure 1B and 1C**) as well as the release of LDH, a marker of lytic cell death (**Figure 1D**). These results indicated that infection with *V. parahaemolyticus* induces the pyroptosis of macrophages, a form of proinflammatory cell death. The processing/release of IL-1β induced by *V. parahaemolyticus* was caspase-1-dependent (**Figure 1B and 1C**), and caspase-1 activation in turn was dependent on ASC, an adaptor protein of NLRs (**Figure 1B and 1C**). Meanwhile, LDH release from infected cells was partly inhibited and delayed in both caspase-1- and ASC-deficient macrophages, suggesting the partial contributions of caspase-1 and ASC in triggering cell death.

The NLR family member proteins NLRP3 and NLRC4 are known to recognize infection with various pathogens and to mediate caspase-1 activation by forming a complex with adaptor protein ASC [1]. To further investigate the mechanisms responsible for caspase-1 activation by *V. parahaemolyticus*, we next examined caspase-1 activation in NLRP3- or NLRC4-deficient macrophages in response to infection with *V. parahaemolyticus*. The caspase-1 activation and IL-1β processing/release were significantly inhibited in NLRP3-deficient BMMs, but not in NLRC4-deficient cells (**Figure 1E and 1F**). In NLRP3-deficient BMMs, a small amount of activated p10 fragment of caspase-1 was still detected. To clarify whether NLRC4 is involved in residual caspase-1 activation in NLRP3-deficient cells, we then generated NLRP3/NLRC4-double deficient mice. Consequently, caspase-1 activation in NLRP3/NLRC4-double deficient macrophages was hardly detected, but the processing and release of IL-1β were not completely inhibited, suggesting that some molecules, such as other NLRs, may trigger weak caspase-1 activation at an almost undetectable level by immunoblotting with anti-caspase-1 antibody. Alternatively, some proteases may be activated to cleave proIL-1β in infected NLRP3/NLRC4 double-deficient macrophages. On the other hand, the release of LDH induced by infection with *V. parahaemolyticus* was partially inhibited in infected NLRP3-deficient cells (**Figure 1G**). Our collective findings from the infection with WT bacteria suggest that NLRP3, but not NLRC4, plays a major role in inducing caspase-1 activation. We also examined caspase-1 activation in infected wild-type and NLRC4-deficient macrophages in the presence of a high concentration of extracellular potassium (**Figure S1A and S1B**), which is known to inhibit NLRP3 inflammasome activation [15]. Interestingly, the processing/release of IL-1β was also almost completely inhibited in KCl-treated NLRC4-deficient cells. Very high concentrations of extracellular potassium also block the activation of NLRP1, NLRC4 and AIM2 inflammasomes [**16**]. Our data may raise the alternative possibility that potassium can block some activities of other NLRs or proteases in NLRC4-deficient macrophages.

### Thermostable direct hemolysins and type III secretion system-1 of *V. parahaemolyticus* are essential for triggering caspase-1 activation

**Figure 2:**
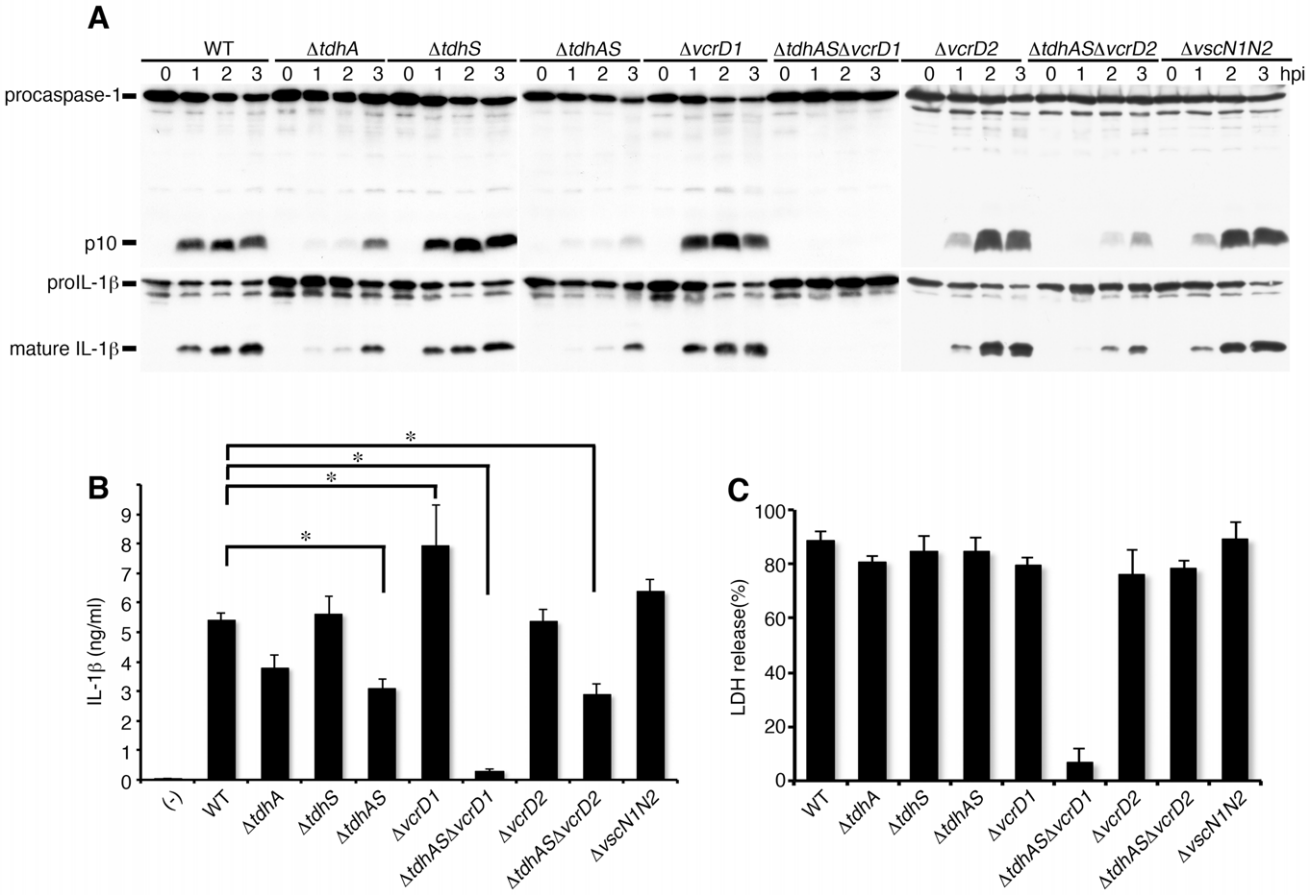
Thermostable direct hemolysins (TdhA and TdhS) and T3SS1 are essential for the induction of caspase-1 activation and cell death. BMMs from wild-type mice were primed with LPS and infected with WT *V. parahaemolyticus* or its respective isogenic mutants. Cell lysates and culture supernatants were harvested at indicated times post infection. **A.** Activation of caspase-1 and IL-1β processing in infected wild-type BMMs were analyzed using immunoblotting with anti-caspase-1 or anti-IL-1β antibody. **B.** IL-1β secretion into culture supernatants from infected BMMs at 3 hpi. was analyzed using an ELISA. Data are presented as the means ± SD of triplicate samples. *p<0.05. **C.** Culture supernatants from infected BMMs were analyzed for LDH release. Data are presented as the means ± SD of triplicate samples.

To identify the bacterial factors that induce caspase-1 activation in infected macrophages, we first focused on the major virulence factors of *V. parahaemolyticus*, thermostable direct hemolysins (TdhA and TdhS) and two type III secretion systems (T3SSs). A series of virulence factor gene-deletion mutants were constructed and their abilities to trigger caspase-1 activation were analyzed. Caspase-1 activation and IL-1β processing were markedly attenuated after infection with the TdhA mutant (Δ*tdhA*) (**Figure 2A and 2B**). The deletion of TdhS did not affect caspase-1 activation. The attenuation of the activation by infection with a double mutant of TdhA and S (Δ*tdhAS*) was observed, but caspase-1 activation and IL-1β processing persisted, suggesting that additional bacterial stimulators are involved in caspase-1 activation. *V. parahaemolyticus* has two T3SS coded in separate loci on its genome [10]. We generated deletion mutants lacking *vcrD1* or *vcrD2*, essential components of T3SS1 or T3SS2, respectively. The single T3SS1 mutant (Δ*vcrD1*) and single T3SS2 mutant (Δ*vcrD2*), as well as a mutant lacking both T3SS1 and T3SS2 (Δ*vscN1N2*), triggered caspase-1 activation and IL-1β processing/release. However, a triple mutant of TdhAS and T3SS1 (Δ*tdhAS*Δ*vcrD1*) did not induce either caspase-1 activation or the processing/release of IL-1β (**Figure 2A and 2B**). By contrast, the Δ*tdhAS*Δ*vcrD2* mutant continued to induce caspase-1 activation. LDH release was completely inhibited only when the cells were infected with Δ*tdhAS*Δ*vcrD1*, suggesting that the lytic cell death of BMMs is dependent upon both TdhAS and T3SS1 (**Figure 2C**). Collectively, these data suggest that TdhAS and the T3SS1 of *V. parahaemolyticus* are essential bacterial factors for activation of caspase-1, IL-1β release and cytotoxicity in infected macrophages.

Notably, we observed the enhancement of IL-1β release after infection with a T3SS1 mutant expressing TdhA and S (Δ*vcrD1*), compared with that after infection with WT bacteria (**Figure 2B**), raising the possibility that T3SS1 is involved in the inhibitory function in addition to triggering caspase-1 activation.

We further examined whether *V. parahaemolyticus* requires an intracellular localization to trigger T3SS1-mediated caspase-1 activation. Cytochalasin D is an inhibitor of phagocytosis that disrupts filamentous actin in host cells and has previously been shown to inhibit pyroptosis induced by *Salmonella* pathogenicity island 1 (SPI-1) [17], [18]. The cytochalasin D treatment inhibited internalization of WT *V. parahaemolyticus* or Δ*tdhAS* as well as *Salmonella* (Figure S2A and S2B). In contrast to the effect seen during *Salmonella* infection, caspase-1 activation was not inhibited by cytochalasin D during infection of BMMs with the WT *V. parahaemolyticus* or Δ*tdhAS* mutant (T3SS1+). Similarly, the stimulation with ATP triggered NLRP3 inflammasome activation in phagocytosis-independent manner (**Figure S2C and S2D**). These data suggest that the phagocytosis of bacteria is not necessary for *V. parahaemolyticus* T3SS1-inducing inflammasome activation.

### TDHs trigger caspase-1 activation via NLRP3 inflammasome, whereas T3SS1 triggers both NLRP3 and NLRC4 inflammasomes

**Figure 3:**
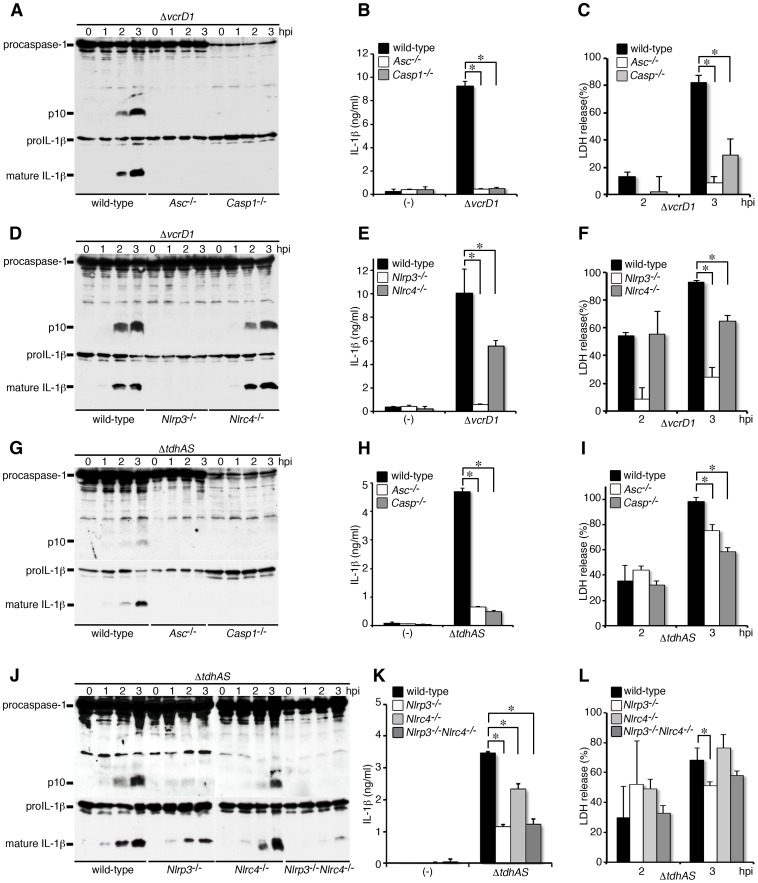
TDHs trigger caspase-1 activation via NLRP3 inflammasome, whereas T3SS1 triggers both NLRP3 and NLRC4 inflammasomes. BMMs from wild-type, ASC-deficient (*Asc^−/−^*), caspase-1-deficient (*Casp1^−/−^*) NLRP3-deficient (*Nlrp3* ^−/−^), NLRC4-deficient (*Nlrc4* ^−/−^), or NLRP3/NLRC4-double deficient (*Nlrp3* ^−/−^ *Nlrc4* ^−/−^) mice were primed with LPS and infected with *V. parahaemolyticus* mutant, Δ*vcrD1* (TDHs intact) or Δ*tdhAS* (T3SS1 intact). **A, D, G and J.** Activation of caspase-1 and IL-1β processing in infected BMMs were analyzed using immunoblotting with anti-caspase-1 or anti-IL-1β antibody. **B, E, H and K.** IL-1β secretion into culture supernatants from infected BMMs at 3 hpi. was analyzed using an ELISA. Data are presented as the means ± SD of triplicate samples. *p<0.05. **C, F, I and L.** Culture supernatants from infected BMMs were analyzed for LDH release. Data are presented as the means ± SD of triplicate samples. *p<0.05.

We next examined the NLR function in caspase-1 activation triggered by TDHs or T3SS1, respectively. Using the Δ*vcrD1* mutant, we examined TDHs-triggered caspase-1 activation and the processing/release of IL-1β in infected wild-type, ASC-, caspase-1-, NLRP3- or NLRC4-deficient BMMs. As shown in **Figure 3A, 3B, 3D, and 3E**, caspase-1 activation and the processing/release of IL-1β were completely inhibited in the infection of NLRP3- and ASC-deficient BMMs, whereas inhibition was marginal in NLRC4-deficient cells. Thus, these results indicate that NLRP3 and ASC are essential host factors for caspase-1 activation induced by TDHs, and also partially are involved in LDH release from infected BMMs (**Figure 3C and 3F**).

The NLRs involved in T3SS-triggering caspase-1 activation were analyzed using Δ*tdhAS* mutant. Caspase-1 activation was apparently abrogated in infected NLRP3-deficient BMMs, but residual cleavage and the release of IL-1β were detected (**Figure 3G, 3H, 3J and 3K**). Wild-type and NLRC4-deficient cells supported T3SS1-mediated caspase-1 activation. In contrast, caspase-1 activation but not IL-1β processing/release was almost suppressed in NLRP3/NLRC4-double deficient BMMs, suggesting that NLRP3 and in part NLRC4 are involved in T3SS1-triggered caspase-1 activation. LDH release was partially dependent on caspase-1, ASC, and NLRP3 (**Figure 3I and 3L**), suggesting that T3SS1-mediated cytotoxicity is in part dependent on NLRP3 inflammasome activation. Consistent with these results, both caspase-1 activation and the processing/release of IL-1β were not detected in KCl-treated NLRC4-deficient BMMs (**Figure S1C and S1D**). The NLRP3/NLRC4-independent weak activation of caspase-1 or caspase-1-independent processing/release of IL-1β may be triggered by T3SS1, and this activity can be blocked by high concentration of extracellular potassium.

### T3SS1 effectors suppress NLRC4-mediated inflammasome activation

**Figure 4:**
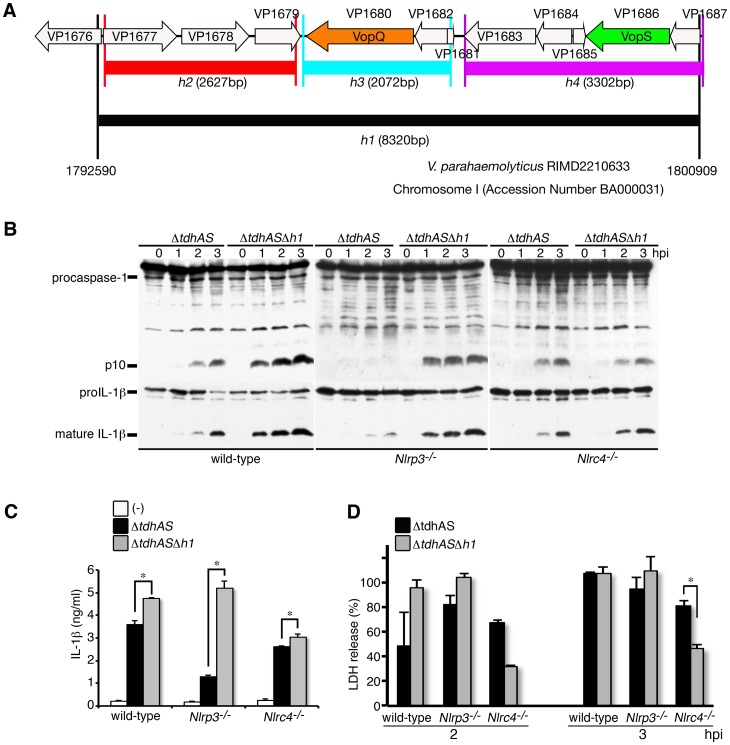
T3SS1 effectors suppress NLRC4-mediated inflammasome activation. BMMs from wild-type, NLRP3-deficient (*Nlrp3* ^−/−^), or NLRC4-deficient (*Nlrc4* ^−/−^) mice were primed with LPS and infected with *V. parahaemolyticus* mutant, Δ*tdhAS* or Δ*tdhAS*Δ*h1*in which the effectors' genes are deleted. **A.** Schematic representation of gene structure in the h1 region of T3SS1. **B.** Activation of caspase-1 and IL-1β processing in infected BMMs were analyzed using immunoblotting with anti-caspase-1 or anti-IL-1β antibody. **C.** IL-1β secretion into culture supernatants from infected BMMs at 3 hpi. was analyzed using an ELISA. Data are presented as the means ± SD of triplicate samples. *p<0.05. **D.** Culture supernatants from infected BMMs were analyzed for LDH release. Data are presented as the means ± SD of triplicate samples. *p<0.05.

The established genome analysis suggests that several T3SS1 effectors are coded in an 8320-bp gene fragment designated as the h1 region in the T3SS1 gene cluster (**Figure 4A**) [19], [20]. The h1 region contains the putative 11 open reading frames including the established 2 effectors (VopQ and VopS) and their chaperons (VP1682 and VP1687, respectively). To examine the relevance of T3SS1 effectors of *V. parahaemolyticus*, the abilities of Δ*tdhAS* or Δ*tdhAS*Δ*h1* mutants to trigger caspase-1 activation were compared. Surprisingly, when the h1 region was deleted, T3SS1-mediated caspase-1 activation and IL-1β processing/release were significantly enhanced (**Figure 4B and 4C**). To clarify whether inflammasome activation via NLRP3 or NLRC4 is enhanced by the deletion, we compared caspase-1 activation in wild-type, NLRP3- or NLRC4-deficient BMMs. The enhancement of caspase-1 activation was remarkable in infected NLRP3-deficient BMMs, compared with the other BMMs (**Figure 4B and 4C**), suggesting that T3SS1 effectors mainly suppress the activation of the NLRC4 inflammasome. The LDH release from wild-type or NLRP3-deficient BMMs was not strongly affected by the h1 deletion (**Figure 4D**). However, LDH release from NLRC4-deficient cells was considerably decreased by infection with Δ*tdhAS*Δ*h1*, raising the possibility that some effectors encoded in the h1 region affect NLRP3-mediated LDH release in a caspase-1 activation-independent manner.

Infection with the Δ*tdhAS*Δ*h1* mutant confirmed that NLRP3/NLRC4-inflammasomes activation is triggered by T3SS1 (**Figure S3**). Notably, caspase-1 activation and processing/release of IL-1β were almost abrogated in NLRP3/NLRC4-double deficient BMMs. The residual processing/release of IL-1β triggered by T3SS1 in NLRP3/NLRC4-double deficient cells (**Figure 3J and 3K**) may result from some functions of the effectors coded in the h1 region, but more detailed mechanisms are unclear.

Furthermore, to examine the functional involvement of *V. parahaemolyticus* flagellin in triggering the NLRC4 inflammasome, we introduced the deletion of two flagellin genes (*lafK* and *flaK*) in the genome of Δ*tdhAS*Δ*h1* mutant [21], [22], [23]. The enhancement of caspase-1 activation and IL-1β processing/release by h1 deletion mutant were significantly decreased by introducing deletions of the flagellin genes in NLRP3-deficient BMMs (**Figure S4**), suggesting that flagellins are predominantly involved in NLRC4 inflammasome activation.

To clarify how h1 deletion affects caspase-1 activation upon infection by *V. parahaemolyticus*, we examined the amounts of released IL-1β and IL-18 from the infected BMMs with or without LPS pretreatment. The released cytokines were significantly enhanced infected with *Δh1* mutant compared with those from BMMs infected with WT bacteria (**Figure S5A and S5B**). The enhancement was observed in LPS priming-independent manner. These results suggest that the effectors coded in the h1 region suppress caspase-1 activation triggered by TDHs or T3SS1 during the infection.

### VopQ and VopS effectors are involved in the suppression of inflammasome activation

**Figure 5:**
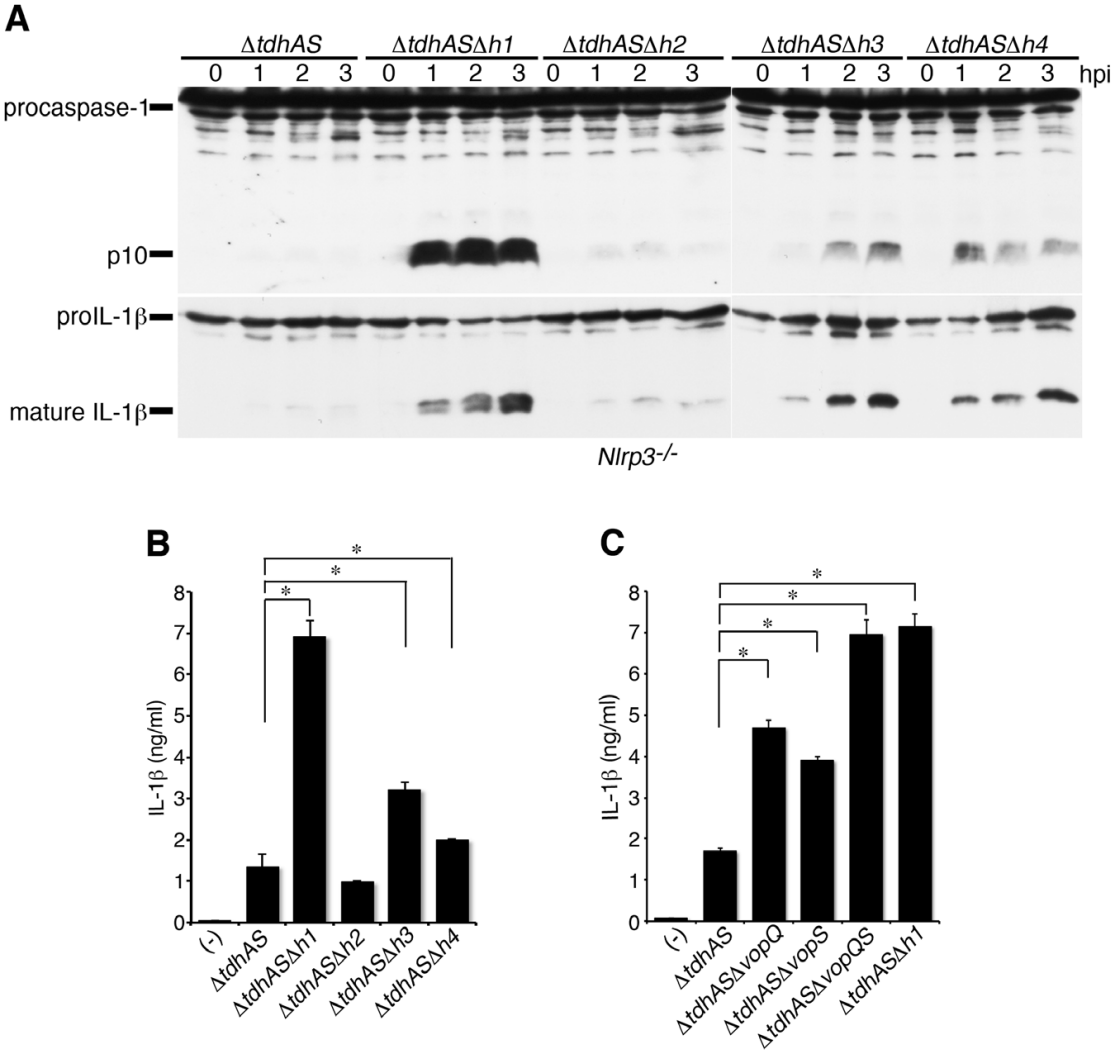
The effectors VopQ and VopS are involved in the suppression of inflammasome activation. **A, B and C.** BMMs from NLRP3-deficient (*Nlrp3* ^−/−^) mice were primed with LPS and infected with Δ*tdhAS* or its respective isogenic mutants as indicated. **A.** Activation of caspase-1 and IL-1β processing in infected BMMs were analyzed using immunoblotting with anti-caspase-1 or anti-IL-1β antibody. **B and C.** IL-1β secretion into culture supernatants from infected BMMs at 3 hpi. was analyzed using an ELISA. Data are presented as the means ± SD of triplicate samples. *p<0.05.

Our data suggest that the T3SS1 effectors coded in the h1 region have inhibitory effects on NLRC4 inflammasome activation. To identify the effector genes, we further introduced a deletion in the h2, h3 or h4 region (**Figure 4A**) and examined NLRC4 inflammasome activation after infection with the h2, h3, or h4 mutants as well as the h1 mutant in NLRP3-deficient BMMs. The deletion of the h2 region did not enhance caspase-1 activation, whereas the deletions of the h3 and h4 regions partially enhanced activation (**Figure 5A and 5B**), suggesting that the effectors coded in the h3 and h4 regions act additively to inhibit the inflammasome activation.

Two effectors of T3SS1, VopQ (also called as VP1680 or VepA) and VopS (VP1686 or VepB) were previously identified in the h3 and h4 regions, respectively (**Figure 4A**). VopQ induces phosphatidylinositol-3-kinase (PI3K)-independent autophagy in infected HeLa cells [24], whereas VopS induces the AMPylation of Rho GTPases such as Cdc42 [25]. Both effectors are thought to be involved in the cell death of infected HeLa cells. We focused on VopQ and VopS and constructed deletion mutants for these effectors in a *ΔtdhAS* background to examine IL-1β release from infected NLRP3-deficient BMMs. The single mutation of VopQ or VopS revealed the partial enhancement of IL-1β secretion (**Figure 5C**). However, when infected with double mutant of VopQS (Δ*tdhAS*Δ*vopQS*), the IL-1β release in infected NLRP3-deficient macrophages was enhanced to a similar extent as that upon infection with Δ*tdh*Δ*h1*. These results suggest that caspase-1 activation-inhibiting factors coded in the h1 region can be attributed to the two effectors, VopQ and VopS.

### VopQ-mediated autophagy interferes with NLRC4 inflammasome activation

**Figure 6:**
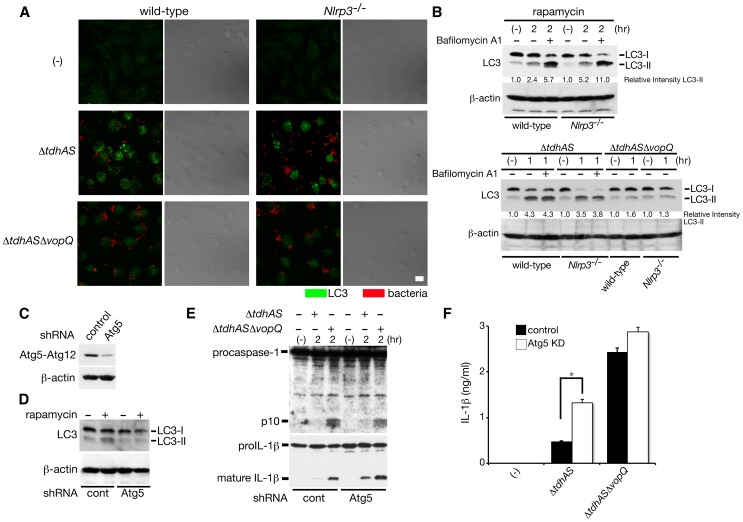
VopQ-mediated autophagy interferes with NLRC4 inflammasome activation. **A.** BMMs from wild-type or NLRP3-deficient (*Nlrp3* ^−/−^) mice were infected with Δ*tdhAS* or Δ*tdhAS*Δ*vopQ* mutants for one hour, and then immunostained with FITC-labeled anti-LC3 antibody (green) and Cy5-labeled anti-*V. parahaemolyticus* antibody (red). The merged image and differential interference contrast (DIC) were shown. Bar, 10 µm. **B.** BMMs were infected with bacteria (1 h) or treated with rapamycin as a control (2 h) in the absence or presence of bafilomycin A1. The cells were analyzed by immunoblotting with anti-LC3 antibody to evaluate the conversion of endogenous LC3-I to II or with anti-β-actin antibody. The relative intensity of LC3-II was calculated by normalizing the untreated control to a relative intensity of 1 and then dividing subsequent LC3-II band intensity by actin band intensity using densitometry. **C.** Knockdown efficiency of Atg5 by shRNA in NLRP3-deficient BMMs was analyzed by immunoblotting with anti-Atg5 antibody. **D.** Effect of knockdown of Atg5 on rapamycin-mediated LC3-I to II conversion in NLRP3-deficient BMMs. **E.** Effect of knockdown of Atg5 on caspase-1 activation and IL-1β processing in infected NLRP3-deficient BMMs infected with indicated bacteria. **F.** Effect of knockdown of Atg5 on IL-1β release from infected NLRP3-deficient BMMs at 3 hpi. was analyzed using an ELISA. Data are presented as the means ± SD of triplicate samples. *p<0.05.

To investigate the effect of VopQ function on the suppression of inflammasome activation, we first examined whether VopQ induce autophagy in infected macrophages. The wild-type or NLRP3-deficient BMMs were infected with *ΔtdhAS* or Δ*tdhAS*Δ*vopQ* and immunostained with anti-LC3 antibody. As shown in **Figure 6A**, VopQ-dependent autophagosome accumulation was induced in infected wild-type and NLRP3-deficient BMMs. Also, the conversion of LC3-I to LC3-II was induced upon infection with *ΔtdhAS* but not with Δ*tdhAS*Δ*vopQ* (**Figure 6B**), suggesting that VopQ-dependent autophagy is induced in both wild-type and NLRP3-deficient BMMs. To further analyze the formation of autophagosomes by VopQ, we examined the conversion of LC3 in infected BMMs with *ΔtdhAS* in the presence of bafilomycin A1, an inhibitor of autophagosome–lysosome fusion [26] according to recent criteria [27]. Unexpectedly, the amounts of LC3-II in both WT and NLRP3-deficient BMMs infected with *ΔtdhAS* were unaffected by bafilomycin A1, in contrast to rapamycin-induced LC3-II production, which was enhanced by the addition of bafilomycin A1 (Figure 6B). These data suggest that VopQ induces autophagosome accumulation by inhibiting autophagic degradation rather than by enhancing autophagic flux. Next, to examine whether VopQ-induced autophagy suppresses the NLRC4 inflammasome, we performed the short hairpin RNA (shRNA) knockdown of ATG5, one of the components of autophagy in NLRP3-deficient BMMs and assessed inflammasome activation upon bacterial infection. By the knockdown of ATG5, the rapamycin-induced conversion of LC3-I to II was down-regulated (**Figure 6C and 6D**). Caspase-1 activation and the processing/release of IL-1β upon infection with *ΔtdhAS* were partially but significantly enhanced by the knockdown of ATG5 (**Figure 6E and 6F**). We also observed a marginal increase in IL-1β release from Atg5-knockdown BMMs compared with that from control cells after infection with Δ*tdhAS*Δ*vopQ*. It is possible that the induction of autophagy by additional factors may be involved to some extent in the regulation of NLRC4 inflammasome activation. These results suggest that VopQ-mediated autophagy interferes with the NLRC4 inflammasome.

On the other hand, infected NLRP3-deficient macrophages treated with rapamycin did not affect either caspase-1 activation or processing/release of IL-1β by bacterial infection which trigger NLRC4 inflammasome (**Figure S6**), suggesting that PI3K-mediated signaling for autophagy is unassociated with the suppression of the NLRC4 inflammasome, consistent with the finding that VopQ induces PI3K-independent autophagy induction [24].

### Inactivation of Cdc42 by VopS is involved in the suppression of NLRC4 inflammasome activation

**Figure 7:**
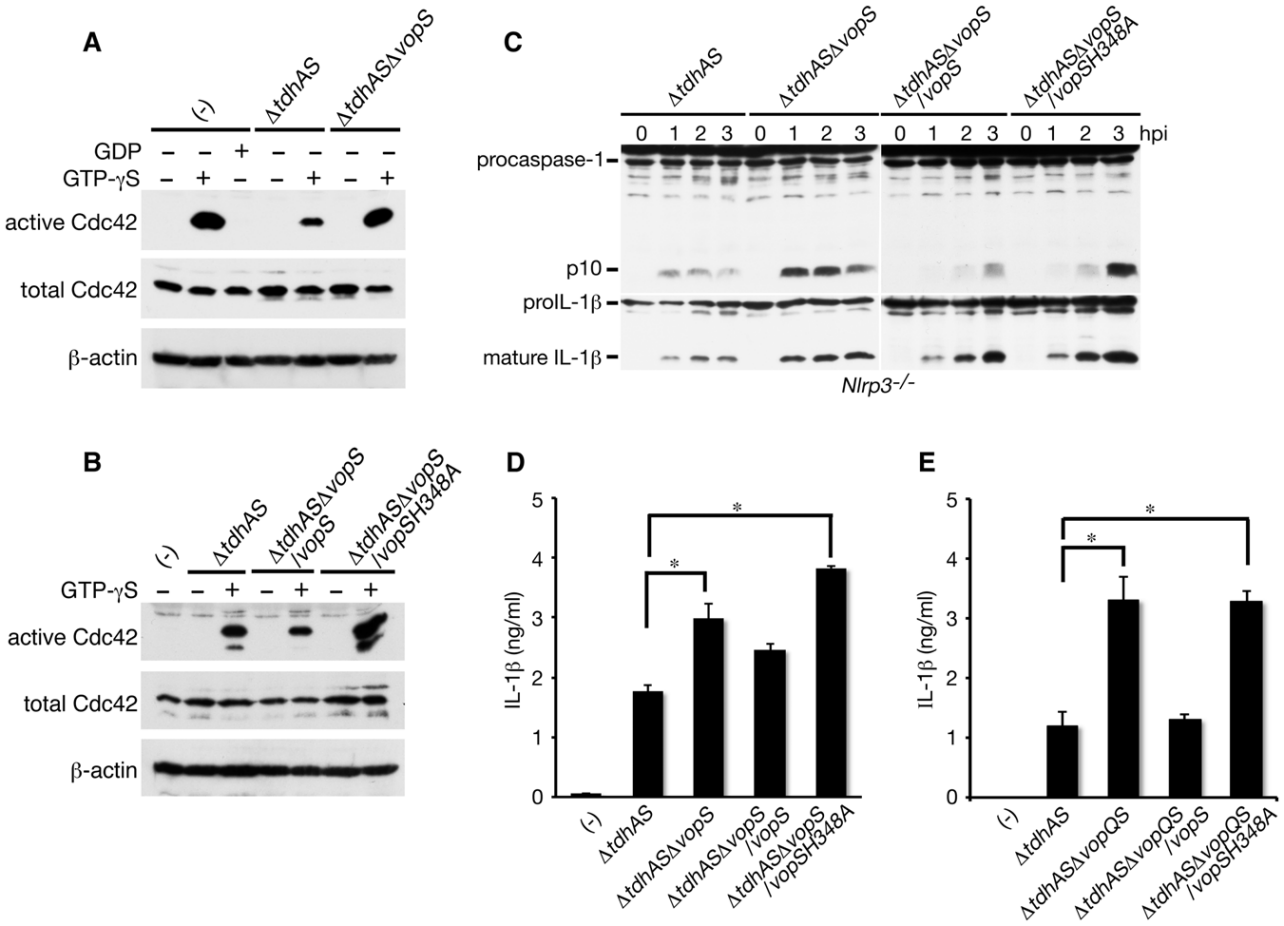
Inactivation of Cdc42 by VopS is involved in suppression of NLRC4 inflammasome activation. **A and B.** Activated endogenous Cdc42 was analyzed by GST-PAK PBD pull-down and immunoblotting with anti-Cdc42 antibody. **A.** The cell lysates of NLRP3-deficient BMMs infected with Δ*tdhAS* or Δ*tdhAS*Δ*vopS* mutants for one hour were loaded with GDP or GTP-γS. **B.** Cell lysates of NLRP3-deficient BMMs infected with Δ*tdhAS*, Δ*tdhAS*Δ*vopS* mutants complemented by WT *vopS* or *vopS* H348A for 1 h were loaded with or without GTP-γS. **C.** Effect of VopS H348A mutant on caspase-1 activation and IL-1β processing in infected NLRP3-deficient BMMs with Δ*tdhAS*Δ*vopS* mutant or complemented strains by wild-type *vopS* or *vopS* H348A. **D.** Effect of VopS H348A mutant on release of IL-1β from infected NLRP3-deficient BMMs with Δ*tdhAS*Δ*vopS* mutant or complemented strains as in **C**. Data are presented as the means ± SD of triplicate samples. *p<0.05. **E.** Effect of VopS H348A mutant on release of IL-1β from infected NLRP3-deficient BMMs with Δ*tdhAS*Δ*vopQS* mutant or complemented strains as in **C**. Data are presented as the means ± SD of triplicate samples. *p<0.05.

To investigate the function of VopS in infected macrophages, we assessed the VopS-mediated inactivation of Rho GTPases such as Cdc42. Since we could not detect the activation of endogenous Cdc42 triggered by infection with *V. parahaemolyticus* using a pull-down assay (**Figure 7A**), the amounts of active Cdc42 in infected BMMs were quantified by loading with GTP-γS followed by a pull-down assay. As shown in **Figure 7A**, the VopS-mediated inactivation of Cdc42 was definitely observed in the cell lysates of infected NLRP3-deficient BMMs. The inactivation of Cdc42 by VopS was confirmed by the transcomplementation of *vopS* gene in the Δ*tdhAS*Δ*vopS* mutant (**Figure 7B**), whereas the inhibitory effect was cancelled by introducing an amino acid substitution H348A in VopS, which is an essential amino acid residue for Rho GTPases inactivation [25]. Using cells infected with the Δ*tdhAS*Δ*vopS* mutant and transcomplemented by wild-type VopS or mutated VopS-H348A, we analyzed the effects of VopS-mediated Cdc42 inactivation on caspase-1 activation and the processing/release of IL-1β in infected NLRP3-deficient BMMs. VopS-H348A, in addition to wild-type VopS, was secreted into the bacterial culture supernatants (data not shown). The wild-type VopS but not VopS-H348A inhibited caspase-1 activation and the processing/release of IL-1β in infected NLRP3-deficient cells (**Figure 7C and 7D**). The VopS-mediated suppression of the NLRC4 inflammasome was more significant when the macrophages were infected with *vopS*-complemented Δ*tdhAS*Δ*vopQS* mutants (**Figure 7E**), probably because of the elimination of the suppression of inflammasome activation by VopQ. These data suggest that VopS-mediated inactivation of Cdc42 (probably also Rac and Rho) is involved in the inhibition of NLRC4 inflammasome activation.

### VopQ and VopS inhibit speck formation by ASC but do not interfere with NLRC4 inflammasome complex formation

To clarify how VopQ or VopS inhibit NLRC4 inflammasome at the molecular level, we first examined the effect of VopQ or VopS on the formation of the NLRC4 inflammasome complex using co-expression in 293T cells and the co-immunoprecipitation of NLRC4 with other components. As shown in **Figure S7A**, and consistent with previous reports [4], [5], the interaction of NAIP2-NLRC4 or NAIP5-NLRC4 was hardly detected, but the co-expression of PrgJ from *Salmonella enterica* serovar Typhimurium significantly enhanced the NAIP2-NLRC4 interaction. Also, the C-terminal portion of FlaA (FlaA-C) of *Legionella pneumophila* enhanced the NAIP5-NLRC4 interaction. We analyzed the proteins that co-precipitated with NLRC4 after infection with the Δ*tdhAS*Δ*vopQ* mutant or the Δ*tdhAS*Δ*vopS* mutant. The infected 293T cells caused cell rounding after 1 hpi in a VopQ or VopS-dependent manner (data not shown), but NLRC4-NAIP2-PrgJ or NLRC4-NAIP5-FlaA-C complex formation was not affected by VopQ or VopS (**Figure S7A**).

We next introduced ASC (as ASC-GFP) to examine the interaction with NLRC4. The co-precipitation of ASC-GFP with NLRC4 also increased in the presence of PrgJ or FlaA-C. However, the ASC-NLRC4 interaction was not affected by infection with the Δ*tdhAS*Δ*vopQ* mutant or the Δ*tdhAS*Δ*vopS* mutant (**Figure S7B**). Consistent with this, the Δ*tdhAS* mutant (expressing both VopQ and S) did not affect NLRC4 inflammasome complex formation (**Figure S7C**). Based on these results, the inflammasome complexes formed by NLRC4 and the other components may not be influenced by VopQ and VopS. However, since the experiments were performed using overexpression systems in 293T cells, it remains possible that VopQ or VopS do block oligomerization physiologically in infected macrophages.

**Figure 8:**
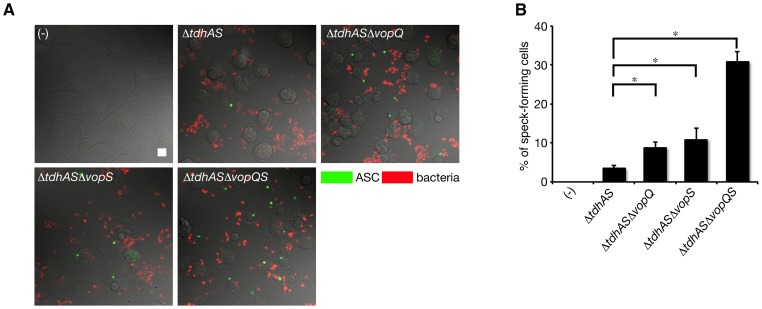
VopQ and VopS inhibit speck formation by ASC. **A.** BMMs from NLRP3-deficient (*Nlrp3* ^−/−^) mice were infected with indicated bacteria for one hour, and then immunostained with FITC-labeled anti-ASC antibody (green) and Cy5-labeled anti-*V. parahaemolyticus* antibody (red). The merged images were shown. Bar, 10 µm. **B.** Quantitative data of number of speck-forming macrophages in **A**. Data are presented as the means ± SD of triplicate samples. *p<0.05.

We next examined speck formation in infected macrophages, a critical step in caspase-1 activation induced by inflammasome activation. NLRP3-deficient BMMs were infected with a series of VopQ or VopS mutants and the specks that formed in the infected cells were analyzed by immunostaining with anti-ASC antibody. Compared with the Δ*tdhAS* mutant, the numbers of speck-forming cells after infection with Δ*tdhAS*Δ*vopQ* mutant or Δ*tdhAS*Δ*vopS* mutant were slightly increased. Furthermore, over 30% of the infected cells contained specks, including ASC, in the absence of VopQ and VopS after infection with Δ*tdhAS*Δ*vopQS* mutant (**Figure 8A and 8B**). These results suggest that VopQ and vopS inhibit speck formation induced by NLRC4 inflammasome activation in infected macrophages but do not interfere with the formation of the NLRC4 inflammasome complex.

## Discussion

A wide variety of human pathogens, including bacteria, trigger caspase-1 activation via NLRP1, NLRP3, NLRC4 or AIM2 inflammasomes. The key factors in the activation of caspase-1 in bacterial infection are mainly pore-forming toxins or virulence-associated secretion systems. Here, we demonstrate that infection with *V. parahaemolyticus* triggers both NLRP3- and NLRC4-inflammasome activation. The pore-forming toxins TdhA and TdhS induce NLRP3 activation, whereas NLRP3/NLRC4 inflammasomes are triggered by T3SS1. TdhA and S have been considered to be the major virulence factors in *V. parahaemolyticus* infection [28]. We provide experimental evidence that these toxins are the bacterial stimulators of NLRP3 inflammasome activation as targets of the innate immune system of the host. In macrophages infected with WT bacteria, TdhA and S play major roles in triggering NLRP3 inflammasome activation. We further found that the T3SS1 effectors VopQ and VopS induce autophagy and the inactivation of Cdc42, respectively, thereby mainly preventing speck formation driven by NLRC4 inflammasome activation.

Several bacterial pathogens have been reported to interfere with inflammasome activation [3]. In most cases, bacteria interfere with the production or recognition of bacterial ligands that trigger inflammasomes. For example, YopK of *Yersinia pseudotuberculosis* blocks the inflammasome from sensing pathogens by interacting with the T3SS translocon [8]. Similarly, in cases with a systemic infection by *Salmonella*, the expressions of flagellin and SPI-1 T3SS are down-regulated to evade the NLRC4 inflammasome, with the converse up-regulating of SPI-2 T3SS [29], [30]. The enzymatic activities of bacterial effectors secreted via T3SS also modulate caspase-1. The phospholipase ExoU, the Rho GTPase-activating protein (GAP) ExoS of *Pseudomonas aeruginosa* [31], [32], the Rho GAP YopE, and the cysteine protease YopT of *Yersinia enterocolitica* negatively regulate caspase-1 activation [33]. However, what NLRs are affected and modulated of inflammasome activation by these bacterial effectors remain to be elucidated. We show that VopQ, a PI3K-independent autophagy inducer, and VopS, a Rho GTPase inactivator of *V. parahaemolyticus*, mostly suppress NLRC4 inflammasome activation. Although the functions of VopQ and VopS in infected macrophages differ, they additively prevent NLRC4 inflammasome activation. Neither VopQ nor VopS interferes with complex formation by NLRC4, NAIP2 (or NAIP5), PrgJ (or flagellin), and the ASC proteins in a co-immunoprecipitation assay. Instead, speck formation in infected macrophages is negatively regulated in a VopQ-dependent and VopS-dependent manner. Specks are also known to be induced by NLRP3 and AIM2 inflammasomes, resulting in the recruitment and aggregation of ASC in the stimulated cells. The reasons why NLRP3 inflammasomes triggered by TDHs or T3SS1 are much less affected than NLRC4 inflammasomes are not yet understood, but we speculate that different signaling mechanisms and functions in the cells support speck formation in response to the activation of different inflammasomes. On the other hand, we cannot exclude the possibility that the inhibition of the NLRC4 inflammasome may result from cell death induced by VopQ or VopS, since these effectors induce cell rounding and the death of infected HeLa cells [24], [25]. It is also possible that the secreted VopQ or VopS may be able to capture the flagellin subunits or rod proteins and down-regulate the activation of NLRC4 inflammasomes. Alternatively, induced autophagosome by VopQ may degrade intracellular flagellin or rod protein delivered via T3SS. Further studies are needed to determine the precise mechanisms responsible for the inhibition of speck formation by VopQ-mediated autophagy induction or VopS-mediated Rho GTPase inactivation.

VopQ induces PI3K-independent autophagy in infected HeLa cells [24]. We demonstrate that VopQ induces autophagosome accumulation by inhibiting autophagic degradation rather than by enhancing autophagic flux, and subsequently inhibits NLRC4 inflammasome-mediated caspase-1 activation. The regulation of inflammasome by autophagy has been recently reported [34], [35]. Basal autophagy prevents the release of mitochondrial DNA sensed by NLRP3 from damaged mitochondria in response to NLRP3 stimulators [34]. Also, the activation of AIM2 or NLRP3 inflammasomes triggers autophagosome formation by recruiting autophagic adaptor p62: conversely, stimulating autophagy limits inflammasome activation [35]. On the other hand, the autophagy induced by VopQ inhibits caspase-1 activation via the NLRC4 inflammasome by preventing speck formation but does not affect the formation of the protein complex, suggesting that the autophagy induced by VopQ acts on later events associated with the NLRC4 inflammasome. Moreover, rapamycin-induced autophagy does not inhibit NLRC4 activation stimulated by *V. parahaemolyticus* or *Salmonella* infection: thus the mTORC1-PI3K pathway driven by rapamycin might not be involved in interference with the NLRC4 inflammasome. Although the manner in which VopQ-mediated autophagosome accumulation prevents speck formation in macrophages is still unexplained, it is most appropriate to speculate that the heterotopic autophagosome accumulation induced by VopQ is able to block the machinery required for speck formation. The further characterization of the VopQ-mediated autophagy signaling pathway may shed light on the mechanisms responsible for the assembly of ASC specks triggered by NLRC4 inflammasome.

The inactivation of Rho GTPase, including Cdc42, by VopS inhibits NLRC4-mediated caspase-1 activation, suggesting that Cdc42 is involved in NLRC4 inflammasome activation. A previous report has shown that the SopE effector secreted via SPI-1 of *Salmonella* triggers caspase-1 activation through the activation of Cdc42 [36], but the molecular mechanisms of Cdc42-mediated inflammasome activation are still largely unknown. In this context, the GAP activity of ExoS or YopE in the inactivation of Rho GTPases is involved in the inhibition of caspase-1 activity, but how GAP activity negatively controls the inflammasome activation remains unknown. We demonstrate that VopS inhibits ASC speck formation, suggesting that the basal activity of Cdc42, Rac or Rho supports the assembly of specks in infected cells. As the activation of endogenous Cdc42 is not observed upon infection with *V. parahaemolyticus*, we speculate that the basal activity of small GTPases is sufficient for maintaining the speck formation. Another explanation for the suppression of inflammasome activation is that the effect of Cdc42 inhibition may cause differential uptake of WT or VopS mutant and results in less contact between bacteria and macrophages, and thus a decrease in total translocation by the T3SS. However, this possibility can be excluded since inflammasome activation by *V. parahaemolyticus* is not affected by cytochalasin D which blocks phagocytosis of macrophages.

In the absence of priming with LPS, caspase-1 activation is readily induced during the early stage of infection with an h1 region deletion mutant in BMMs, suggesting that VopQ and VopS are capable of partially suppressing caspase-1 activation in wild-type bacterial infections leading to both NLRP3 and NLRC4 inflammasomes. The biological importance of effector-mediated NLRC4 suppression during in vivo infection with *V. parahaemolyticus* remains to be elucidated because of the lack of an oral infection model in mice capable of evoking bacterial colonization and inflammation in intestine. We are attempting to establish an infection model in mice. A recent report has shown that intestinal mononuclear phagocytes in the mouse colon predominantly express NLRC4, but not NLRP3, and do not respond to TLR ligands [37]. In these cells, the NLRC4 inflammasome is activated upon infection with *Salmonella*, whereas the activation of NLRP3 inflammasome is not triggered by the NLRP3 stimulators such as ATP [37]. These finding lead us to consider what cell lineages are involved in caspase-1 activation upon infection with *V. parahaemolyticus* in vivo. In in vitro studies using BMMs infected with WT *V. parahaemolyticus*, our results suggest that NLRP3 plays a major role in inducing caspase-1 activation, whereas NLRC4 plays a minor role. However, this conclusion may be limited to particular cell lines, such as BMMs that express both NLRP3 and NLRC4. In in vivo infection, the bacteria are thought to colonize the epithelia of the intestine and to cause epithelial injury through the secretion of TDHs or T3SS-mediated effectors. In such situations, the bacteria are likely exposed to attack from intestinal mononuclear phagocytes that express NLRC4 but not NLRP3 [37], and the inhibition of NLRC4 inflammasome by VopQ and VopS may confer an advantage to invading *V. parahaemolyticus*. Although establishing the biological significance of the VopQ and VopS-mediated inhibition of the NLRC4 inflammasome will require further studies, the elucidation of effector-based suppression of inflammasome activation may provide important insights into bacterial strategies for evading inflammasome-mediated host immune responses.

## Materials and Methods

### Ethics statement

All animal studies were carried out in strict accordance with the Guidelines for Animal Experimentation of the Japanese Association for Laboratory Animal Science. The protocols were approved by the Animal Care and Use Committee of the University of the Ryukyus, Okinawa, Japan (Permit Number: 5350 and 5351).

### Bacterial strains and plasmids

The wild-type *V. parahaemolyticus* strain RIMD2210633 (KP positive, serotype O3:K6) was clinical isolate [38]. Isogenic *V. parahaemolyticus* mutants were constructed using allele replacement strategies and the suicide vector pYAK1 [14]. For complementation in bacteria, the shuttle vector pSA19Cm-MCS was used as described previously [19]. The site directed mutagenesis was performed using QuikChange site-directed mutagenesis kit (Stratagene). The wild-type *Salmonella enterica* serovar Typhimurium SL1344 was described previously [14]. The expression plasmids of FLAG-NLRC4, HA-NAIP2, HA-NAIP5, Myc-PrgJ or Myc-FlaA (C-terminal truncated) were kindly provided from Dr. Shao (National Institute of Biological Sciences, Beijing, China). For expression of ASC-GFP, ASC gene was cloned into pEGFP-N1 (Clontech).

### Mice and preparation of macrophages

C57BL/6 mice were purchased from Japan SLC (Tokyo, Japan) as wild-type mice. C57BL/6 background caspase-1-deficient [39], NALP3-deficient (*Nlrp3* ^−/−^) [40], NLRC4-deficient (*Nlrc4* ^−/−^) [41] and ASC (*Asc* ^−/−^ or *Pycard* ^−/−^)-deficient mice [42] were housed in a pathogen-free facility. Caspase-1-deficient mice also lack caspase-11 [43]. Mice doubly deficient in NLRP3 and NLRC4 (*Nlrp3* ^−/−^ *Nlrc4* ^−/−^) were generated from the single gene-deficient mice. BMMs were prepared from the femurs and tibias of the above mice and cultured for 5–6 days in 10% FCS-RPMI 1640 supplemented with 30% mouse L-cell supernatant.

### Reagents

The ultrapure LPS and cytochalasin D were purchased from Invivogen and Sigma-Aldrich, respectively. Rapamycin was from LC Laboratories. The following antibodies were obtained commercially: rabbit anti-mouse caspase-1 (sc-514, Santa Cruz), goat anti-mouse IL-1β (AF-401-NA, R & D Systems), rabbit anti-mouse IL-18 (5180R-100, BioVision), rabbit anti-mouse LC3 (PM036 and M152-3, MBL), rabbit anti-mouse ATG5 (#8540, Cell Signaling), rabbit anti-mouse Cdc42 (21010, New East Biosciences), rabbit anti-FLAG M2 (F3165, Sigma), mouse anti-HA (MMS-101P, Covance), rabbit anti-Myc tag (#2278, Cell Signaling), anti-*V. parahaemolyticus* (O3, Eiken Chemical), and anti-*S. enterica* (multiple O-antigens, Eiken Chemical). The rabbit anti-VopS antibody and rat anti-mouse ASC antibody were generated previously [44], [45].

### Bacterial infection, LDH assay and ELISA

BMMs were seeded at 5×10^5^ cells in 24-well plates containing 10% FCS-RPMI 1640 and primed with LPS (1 µg/ml) for 3 h to induce the expression of proIL-1β prior to bacterial infection. The cells were infected with *V. parahaemolyticus* grown to mid-log phase at multiplicity of infection (MOI) of ∼10 per cell. The plates were centrifuged at 600 *g* for 10 min to synchronize the stage of infection and incubated. At the times indicated after infection without antibiotic treatment, the lactate dehydrogenase (LDH) activity of the culture supernatants of infected cells was measured by using a CytoTox 96 assay kit (Promega) according to the manufacturer's protocol. The cytokines released in culture supernatants were quantified by ELISA (R &D systems). The end point of time course experiments was limited as 3 hours post infection (hpi), since *Vibrio* are extracellular pathogen and multiply fast in the cell culture media.

### Immunoblot

BMMs were seeded at a density of 2×10^6^ cells per well in 6-well plates and infected with bacteria. The cells were lysed and combined with the supernatant precipitated with 10% trichloroacetic acid. The samples were loaded onto 15% SDS-PAGE, and the cleaved form of caspase-1, IL-1β, was detected using anti-caspase-1, anti-IL-1β or anti-IL-18 antibody, respectively. To compare the intensity of the bands on immunoblots, band density was analyzed using ImageJ densitometry software.

### Immunostaining

For immunofluorescence study, the infected cells were fixed and immunostained as described previously [46], and they were analyzed with a confocal laser-scanning microscope (TCS-SPE, Leica-Microsystems).

### Knockdown of Atg5 in BMMs by shRNA

We used lentiviral pLKO.1-puro vector coding shRNA targeting Atg5, CCGGCCTTGGAACATCACAGTACATCTCGAGATGTACTGTGATGTTCCAAGGTTTTTG (RCN0000099431, Sigma) and negative control vector including non-target shRNA (SHC002, Sigma). Each plasmid was transfected together with two packaging plasmids (pMISSION GAG POL and pMISSION VSV-G, Sigma) into 293T cells. The packaging and lentiviral infection were performed following the manufacturer's instruction. Lentivirus expressing shRNA was collected and infected to BMMs. The puromycin resistant cells were used in bacterial infection. Knockdown efficiency was examined by immunoblotting analysis.

### GTPase activation assay using GST-pull-down

The cells were washed once in cold TBS and samples were collected by scraping into lysis/wash buffer (50 mM Tris-HCl, pH 8.0, 150 mM NaCl, 10 mM MgCl_2_, 1 mM EDTA, 1% Triton X-100) supplemented with a protease inhibitor mixture (Roche). The precleared samples were used for loading with GDP (1 mM) or GTP-γS (0.1 mM) for 30 min at 30°C. GST-pull-downs were performed with 5 µg of purified GST-PAK PBD (Thermo Scientific) on glutathione beads for one hour at 4°C and then washed three times in lysis/wash buffer. The samples on the beads were boiled in SDS sample buffer for 5 min.

### Immunoprecipitation

293T cells were transfected with indicated plasmids. Cells were harvested and lysed in a buffer containing 50 mM Tris-HCl, pH 7.5, 150 mM NaCl and 1% Triton X-100 supplemented with a protease inhibitor mixture. The precleared lysates were subjected to anti-FLAG M2 immunoprecipitation by following the manufacturer's instruction. The beads were boiled in SDS sample buffer followed by immunoblotting analysis.

### Statistical analysis

All data are presented as the mean and standard deviation of at least three determinations per experimental condition. All experiments were performed at least three times and representative results are shown in the figures. Statistical analyses were performed using unpaired two-tailed Student's *t* tests. Differences were considered significant at a p value of <0.05.

## Supporting Information

Figure S1 **Wild-type** ***V. parahaemolyticus*** **- or T3SS-1-induced caspase-1 activation and IL-1β release are almost inhibited in NLRC4-deficient BMMs in the presence of high concentration of KCl.** LPS-primed BMMs from wild-type, NLRP3-deficient (*Nlrp3* ^−/−^), or NLRC4-deficient (*Nlrc4* ^−/−^) mice were infected with wild-type *V. parahaemolyticus* (Vp) or Δ*tdhAS* (T3SS-1+) mutant in the absence or presence of KCl (130 mM). **A and C.** The activation of caspase-1 and IL-1β processing in BMMs were analyzed using immunoblotting with anti-caspase-1 or anti-IL-1β antibody. **B and D.** IL-1β secretion from the infected BMMs into the culture supernatants at 3 hpi. was analyzed using an ELISA. Data are presented as the means ± SD and compared using the unpaired two-tailed Student's *t* test (*p<0.05).
(TIF)

Figure S2 **Phagocytosis of** ***V. parahaemolyticus*** **by macrophages is not necessary for caspase-1 activation triggered by T3SS-1.** **A.** LPS-primed WT BMMs were treated with cytochalasin D for 30 min and then infected with *V. parahaemolyticus* (MOI 10) for 40 min or *Salmonella* (MOI 50) for 20 min. Extracellular bacteria were stained with TRITC-labeled antibodies, which were added before permeabilization with saponin; total bacteria were stained with FITC-labeled antibodies. Merged images with intracellular (green) and extracellular (yellow) bacteria visualized by differential interference contrast are shown. Bar, 10 µm. **B.** Quantitative data showing the number of macrophages containing more than five intracellular bacteria in **A**. Data are mean ± SD of triplicate samples. *p<0.05. **C.** Activation of caspase-1 and IL-1β processing in LPS-primed BMMs was analyzed 1.5 h after *V. parahaemolyticus* infection in the presence of cytochalasin D. Uninfected cells were also incubated during the course of infection. **D.** Activation of caspase-1 and IL-1β processing in BMMs was analyzed after *Salmonella* infection (1.5 hpi) or ATP treatment (5 mM, 30 min) in the presence of cytochalasin D.
(TIF)

Figure S3 **Caspase-1 activation by T3SS-1 is triggered via NLRP3 and NLRC4 inflammasomes.** BMMs from wild-type, NLRP3-deficient (*Nlrp3* ^−/−^), NLRC4-deficient (*Nlrc4* ^−/−^), or NLRP3/NLRC4-double deficient (*Nlrp3* ^−/−^ *Nlrc4* ^−/−^) mice were primed with LPS (1 µg/ml; 3 hr) and infected with Δ*tdhAS*Δ*h1* mutant. **A.** The activation of caspase-1 and IL-1β processing in infected BMMs were analyzed using immunoblotting with anti-caspase-1 or anti-IL-1β antibody. **B.** IL-1β secretion from the infected BMMs into the culture supernatants at 3 hpi. was analyzed using an ELISA. Data are presented as the means ± SD of triplicate samples. *p<0.05.
(TIF)

Figure S4 **Flagellins are major components for triggering NLRC4 inflammasome activation by T3SS-1 of** ***V. parahaemolyticus*** **.** BMMs from wild-type, NLRP3-deficient (*Nlrp3* ^−/−^), or NLRC4-deficient (*Nlrc4* ^−/−^) mice were primed with LPS (1 µg/ml; 3 hr) and infected with indicated mutants for 3 hr. **A.** The activation of caspase-1 and IL-1β processing in infected BMMs were analyzed using immunoblotting with anti-caspase-1 or anti-IL-1β antibody. **B.** IL-1β secretion from the infected BMMs into the culture supernatants was analyzed using an ELISA. Data are mean ± SD of triplicate samples. *p<0.05.
(TIF)

Figure S5 **The T3SS-1 effectors coded in h1 region prevent inflammasome activation.** Wild-type BMMs were incubated with or without LPS for 3 hr and infected with WT *V. parahaemolyticus* or Δ*h1* mutant. **A.** The secretion of IL-1β from the infected BMMs into the culture supernatants was analyzed using an ELISA. Data are mean ± SD of triplicate samples. *p<0.05. **B.** The secretion of IL-18 from the infected BMMs into the culture supernatants was analyzed using an ELISA. Data are mean ± SD of triplicate samples. *p<0.05.
(TIF)

Figure S6 **Rapamycin does not alter NLRC4 inflammasome activation by NLRC4-triggering bacteria.** LPS-primed NLRP3-deficient BMMs were treated with DMSO (-) or rapamycin (25 µg/ml, 2 hr), and treated with ATP (30 min) or infected with *Salmonella* (30 min), *V. parahaemolyticus* Δ*tdhAS* mutant (2 hr) or Δ*tdhAS*Δ*vopQ* mutant (2 hr). **A.** The cells were analyzed by immunoblot for caspase-1 activation and processing of IL-1β. **B.** IL-1β secretion from BMMs into culture supernatants was analyzed by ELISA. Data are mean ± SD of triplicate samples.
(TIF)

Figure S7 **VopQ and VopS do not interfere with NLRC4 inflammasome complex formation.** Cell lysates from 293T cells transfected with the indicated plasmid combinations and infected with Δ*tdhAS*Δ*vopQ*, Δ*tdhAS*Δ*vopS*, or Δ*tdhAS* mutants were subjected to co-immunoprecipitation with anti-FLAG antibody. **A.** Effects of VopQ and VopS on PrgJ-NAIP2-NLRC4 and FlaA-NAIP5-NLRC4 interaction. **B.** Effects of VopQ and VopS on PrgJ-NAIP2-NLRC4-ASC and FlaA-NAIP5-NLRC4-ASC interaction. **C.** Effects of VopQ and VopS using Δ*tdhAS* infection on PrgJ-NAIP2-NLRC4-ASC and FlaA-NAIP5-NLRC4-ASC interaction.
(TIF)
