# Supplementary figures and images for: Vibrio parahaemolyticus Effector Proteins Suppress Inflammasome Activation by Interfering with Host Autophagy Signaling
Source: PLoS Pathog. 2013 Jan 24;9(1):e1003142. doi: 10.1371/journal.ppat.1003142 (PMC3554609; doi:10.1371/journal.ppat.1003142)

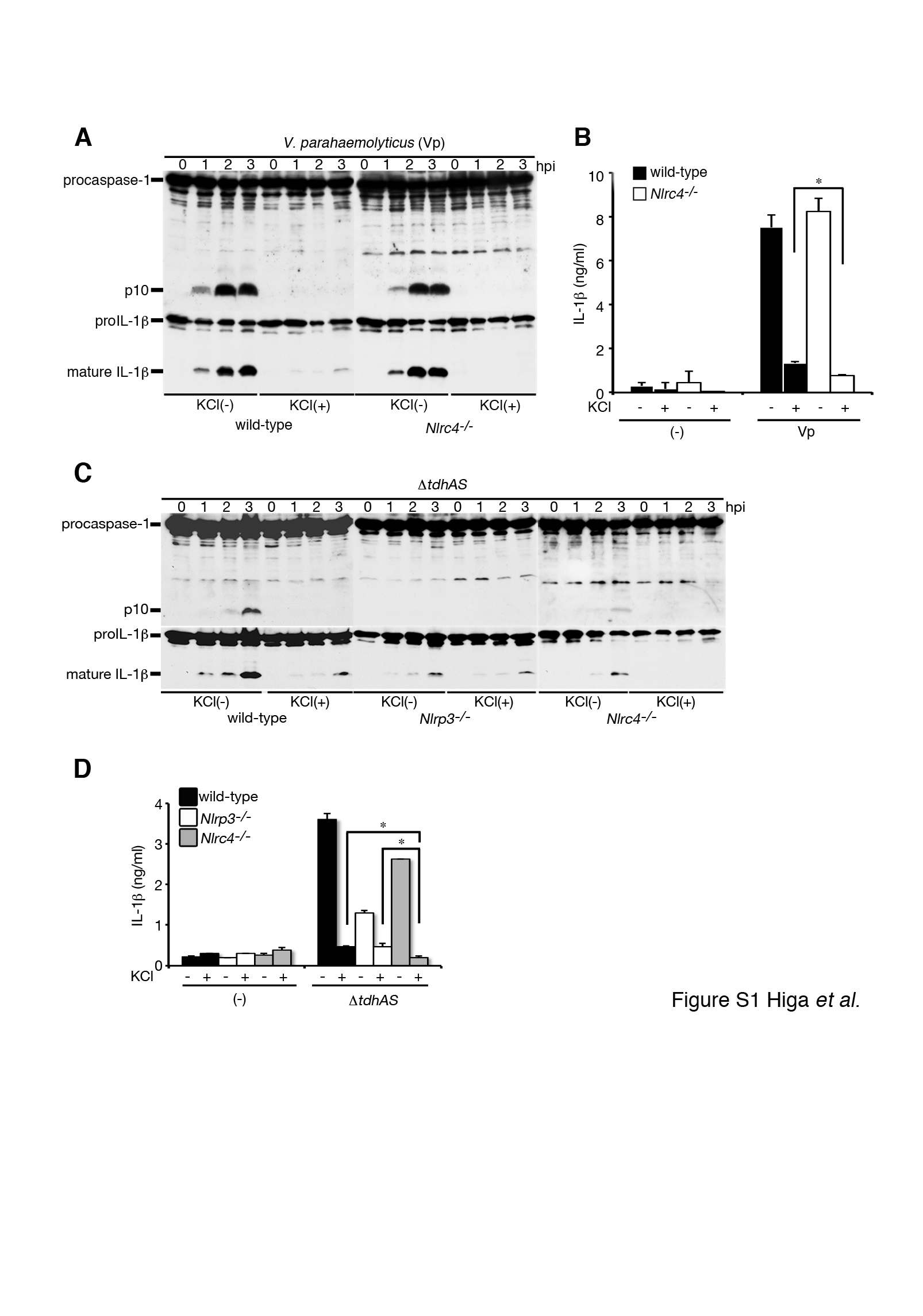

Supplement: Figure S1 — Wild-type V. parahaemolyticus - or T3SS-1-induced caspase-1 activation and IL-1β release are almost inhibited in NLRC4-deficient BMMs in the presence of high concentration of KCl. LPS-primed BMMs from wild-type, NLRP3-deficient (Nlrp3 −/−), or NLRC4-deficient (Nlrc4 −/−) mice were infected with wild-type V. parahaemolyticus (Vp) or ΔtdhAS (T3SS-1+) mutant in the absence or presence of KCl (130 mM). A and C. The activation of caspase-1 and IL-1β processing in BMMs were analyzed using immunoblotting with anti-caspase-1 or anti-IL-1β antibody. B and D. IL-1β secretion from the infected BMMs into the culture supernatants at 3 hpi. was analyzed using an ELISA. Data are presented as the means ± SD and compared using the unpaired two-tailed Student's t test (*p<0.05). (TIF) [file ppat.1003142.s001.tif]

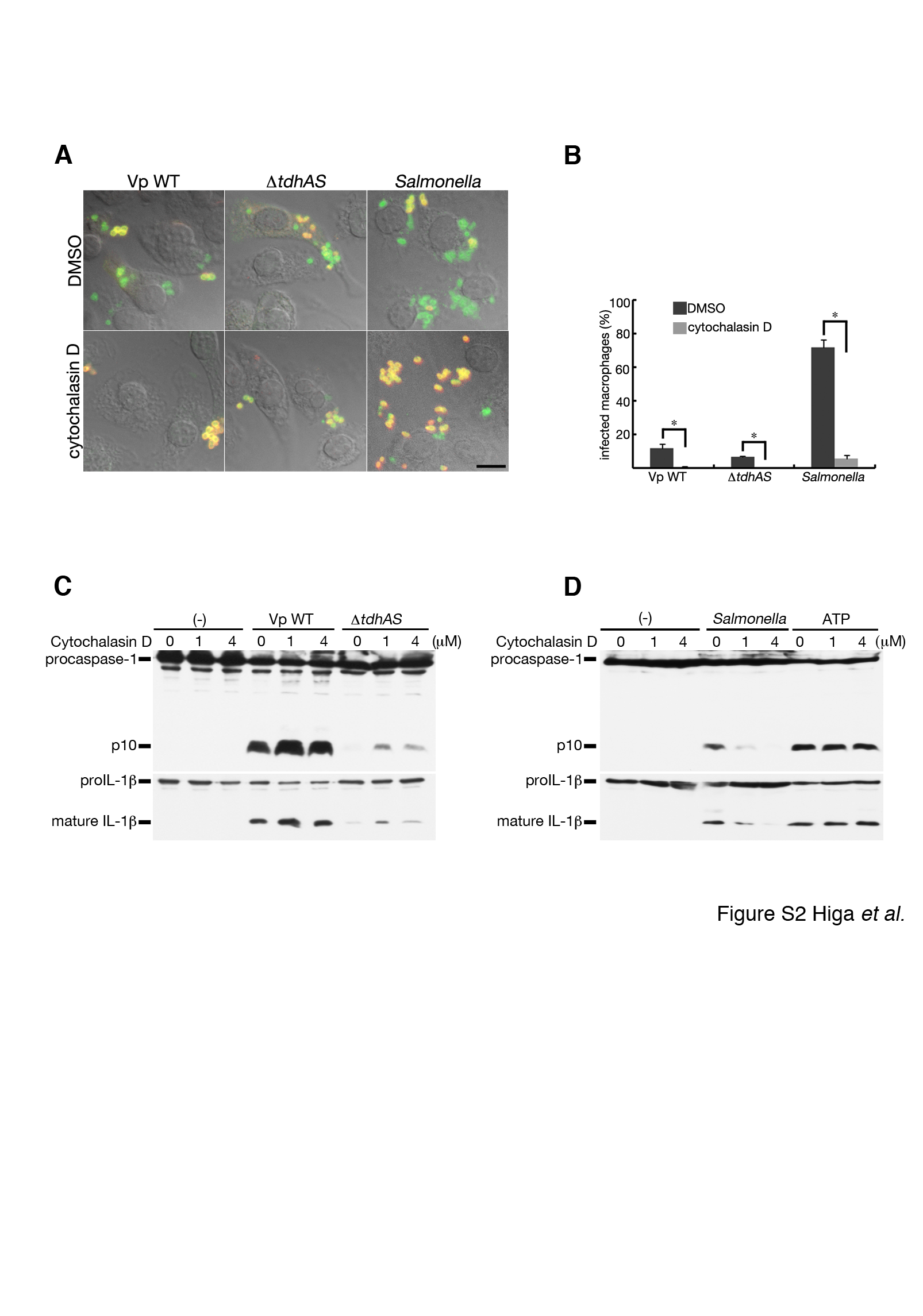

Supplement: Figure S2 — Phagocytosis of V. parahaemolyticus by macrophages is not necessary for caspase-1 activation triggered by T3SS-1. A. LPS-primed WT BMMs were treated with cytochalasin D for 30 min and then infected with V. parahaemolyticus (MOI 10) for 40 min or Salmonella (MOI 50) for 20 min. Extracellular bacteria were stained with TRITC-labeled antibodies, which were added before permeabilization with saponin; total bacteria were stained with FITC-labeled antibodies. Merged images with intracellular (green) and extracellular (yellow) bacteria visualized by differential interference contrast are shown. Bar, 10 µm. B. Quantitative data showing the number of macrophages containing more than five intracellular bacteria in A. Data are mean ± SD of triplicate samples. *p<0.05. C. Activation of caspase-1 and IL-1β processing in LPS-primed BMMs was analyzed 1.5 h after V. parahaemolyticus infection in the presence of cytochalasin D. Uninfected cells were also incubated during the course of infection. D. Activation of caspase-1 and IL-1β processing in BMMs was analyzed after Salmonella infection (1.5 hpi) or ATP treatment (5 mM, 30 min) in the presence of cytochalasin D. (TIF) [file ppat.1003142.s002.tif]

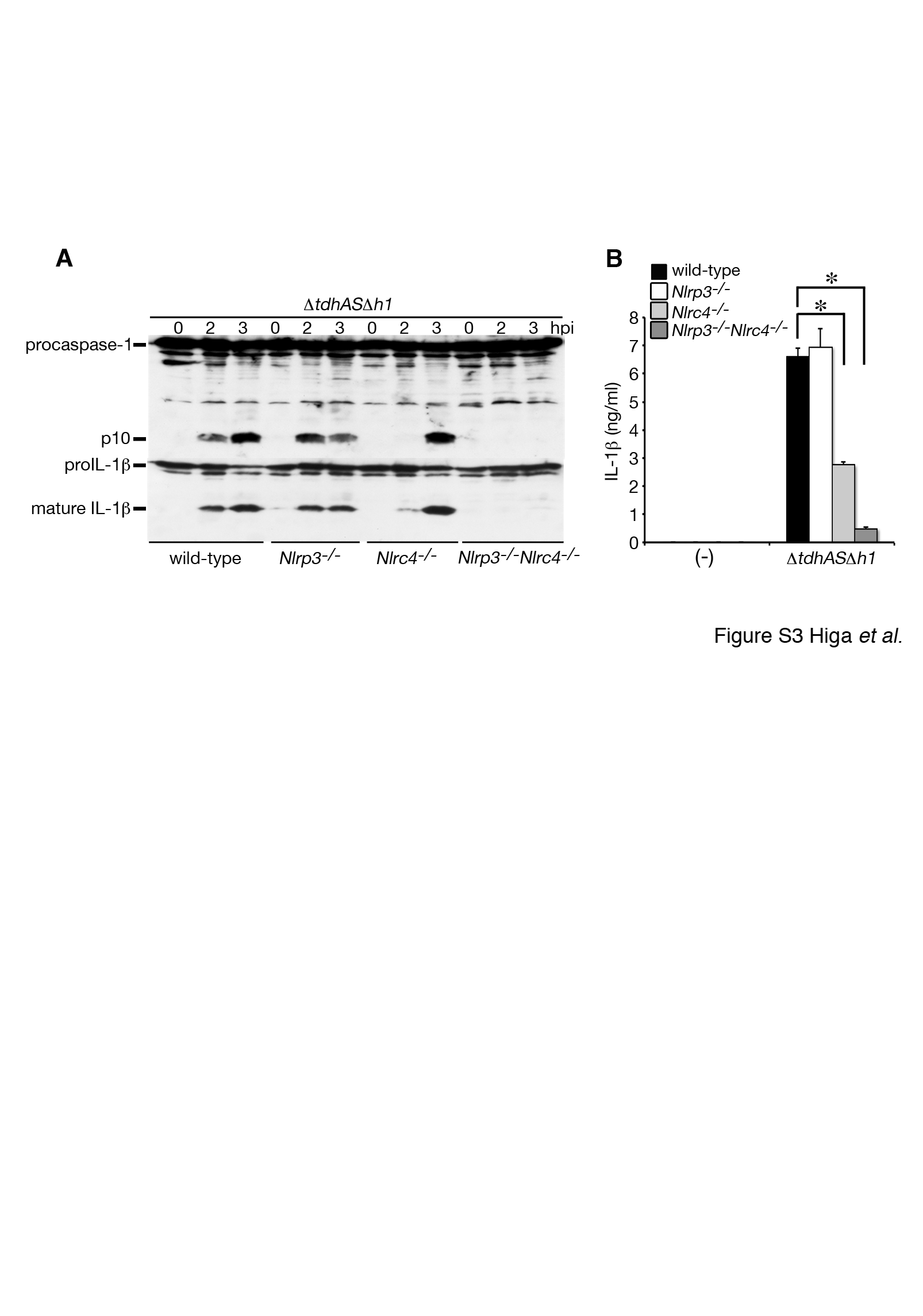

Supplement: Figure S3 — Caspase-1 activation by T3SS-1 is triggered via NLRP3 and NLRC4 inflammasomes. BMMs from wild-type, NLRP3-deficient (Nlrp3 −/−), NLRC4-deficient (Nlrc4 −/−), or NLRP3/NLRC4-double deficient (Nlrp3 −/− Nlrc4 −/−) mice were primed with LPS (1 µg/ml; 3 hr) and infected with ΔtdhASΔh1 mutant. A. The activation of caspase-1 and IL-1β processing in infected BMMs were analyzed using immunoblotting with anti-caspase-1 or anti-IL-1β antibody. B. IL-1β secretion from the infected BMMs into the culture supernatants at 3 hpi. was analyzed using an ELISA. Data are presented as the means ± SD of triplicate samples. *p<0.05. (TIF) [file ppat.1003142.s003.tif]

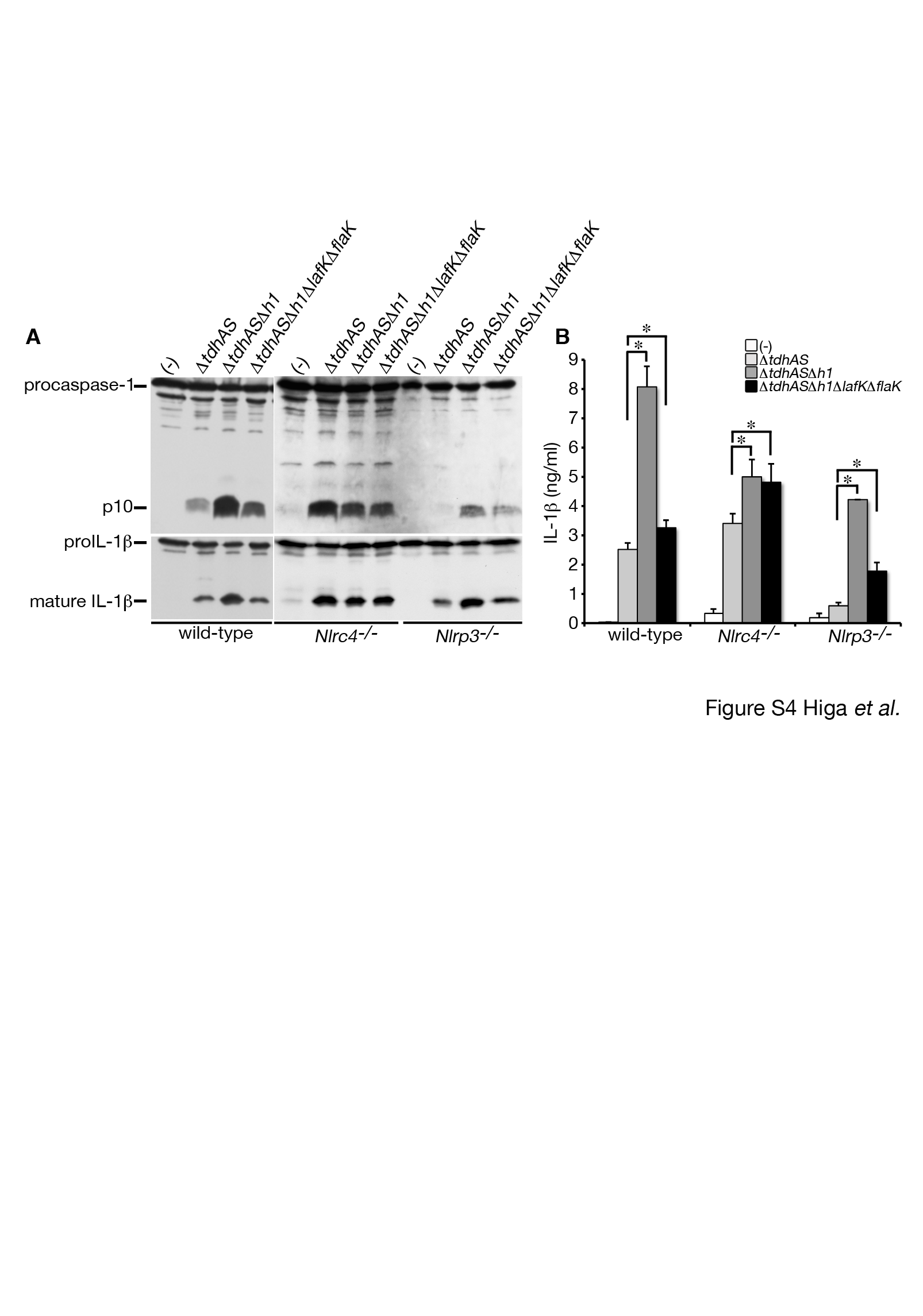

Supplement: Figure S4 — Flagellins are major components for triggering NLRC4 inflammasome activation by T3SS-1 of V. parahaemolyticus . BMMs from wild-type, NLRP3-deficient (Nlrp3 −/−), or NLRC4-deficient (Nlrc4 −/−) mice were primed with LPS (1 µg/ml; 3 hr) and infected with indicated mutants for 3 hr. A. The activation of caspase-1 and IL-1β processing in infected BMMs were analyzed using immunoblotting with anti-caspase-1 or anti-IL-1β antibody. B. IL-1β secretion from the infected BMMs into the culture supernatants was analyzed using an ELISA. Data are mean ± SD of triplicate samples. *p<0.05. (TIF) [file ppat.1003142.s004.tif]

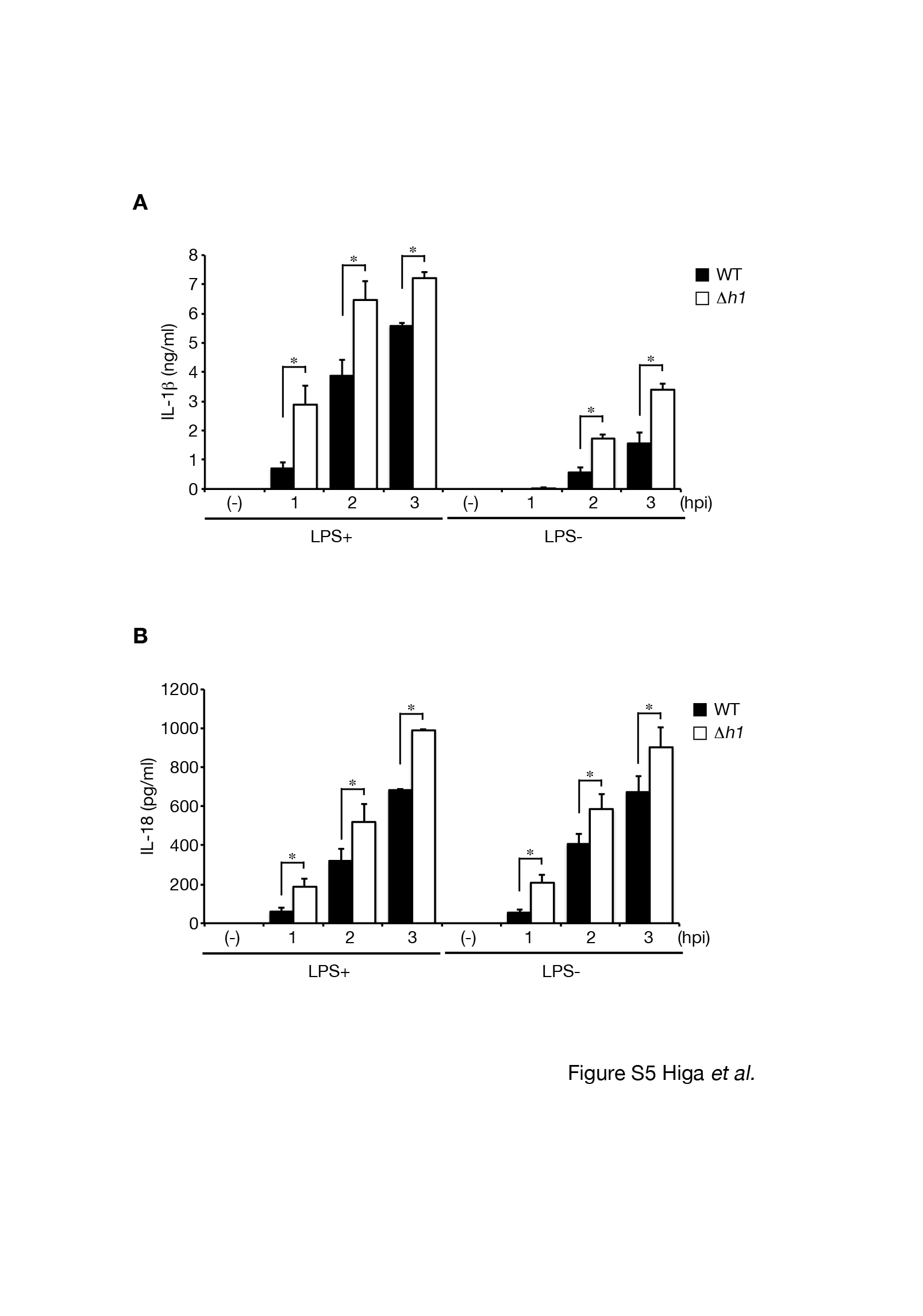

Supplement: Figure S5 — The T3SS-1 effectors coded in h1 region prevent inflammasome activation. Wild-type BMMs were incubated with or without LPS for 3 hr and infected with WT V. parahaemolyticus or Δh1 mutant. A. The secretion of IL-1β from the infected BMMs into the culture supernatants was analyzed using an ELISA. Data are mean ± SD of triplicate samples. *p<0.05. B. The secretion of IL-18 from the infected BMMs into the culture supernatants was analyzed using an ELISA. Data are mean ± SD of triplicate samples. *p<0.05. (TIF) [file ppat.1003142.s005.tif]

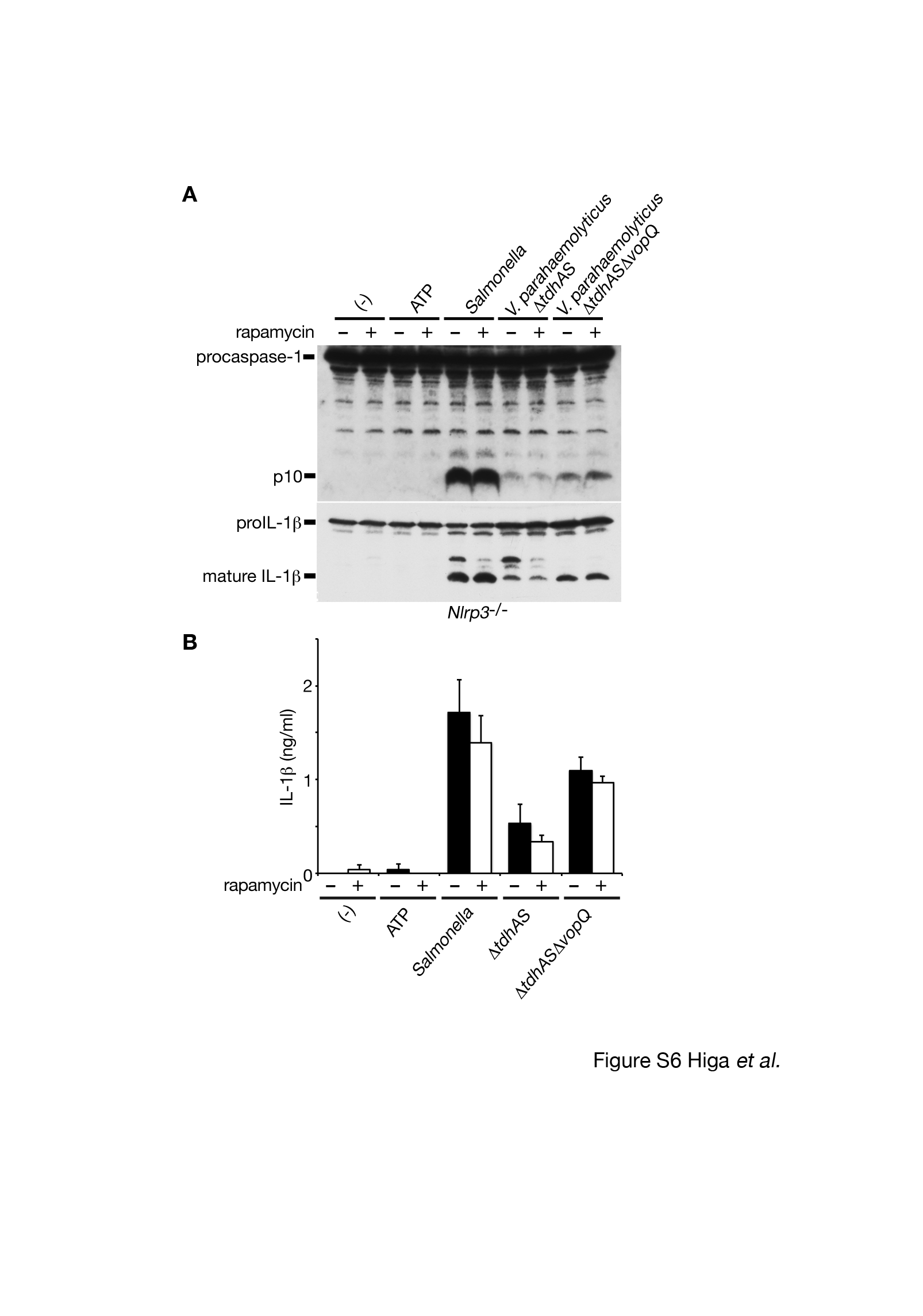

Supplement: Figure S6 — Rapamycin does not alter NLRC4 inflammasome activation by NLRC4-triggering bacteria. LPS-primed NLRP3-deficient BMMs were treated with DMSO (-) or rapamycin (25 µg/ml, 2 hr), and treated with ATP (30 min) or infected with Salmonella (30 min), V. parahaemolyticus ΔtdhAS mutant (2 hr) or ΔtdhASΔvopQ mutant (2 hr). A. The cells were analyzed by immunoblot for caspase-1 activation and processing of IL-1β. B. IL-1β secretion from BMMs into culture supernatants was analyzed by ELISA. Data are mean ± SD of triplicate samples. (TIF) [file ppat.1003142.s006.tif]

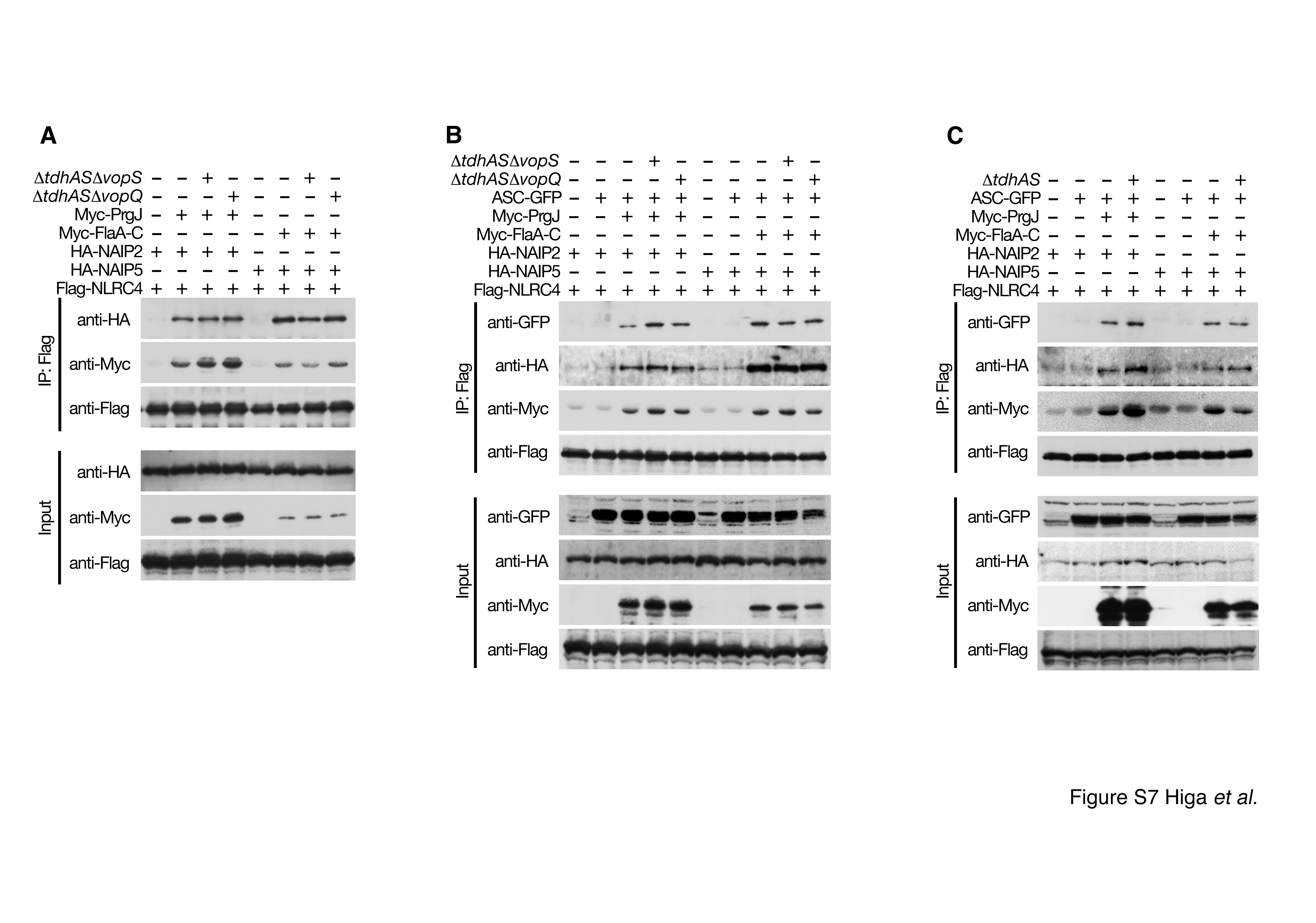

Supplement: Figure S7 — VopQ and VopS do not interfere with NLRC4 inflammasome complex formation. Cell lysates from 293T cells transfected with the indicated plasmid combinations and infected with ΔtdhASΔvopQ, ΔtdhASΔvopS, or ΔtdhAS mutants were subjected to co-immunoprecipitation with anti-FLAG antibody. A. Effects of VopQ and VopS on PrgJ-NAIP2-NLRC4 and FlaA-NAIP5-NLRC4 interaction. B. Effects of VopQ and VopS on PrgJ-NAIP2-NLRC4-ASC and FlaA-NAIP5-NLRC4-ASC interaction. C. Effects of VopQ and VopS using ΔtdhAS infection on PrgJ-NAIP2-NLRC4-ASC and FlaA-NAIP5-NLRC4-ASC interaction. (TIF) [file ppat.1003142.s007.tif]
